# Immunophenotyping of peripheral blood in NSCLC patients discriminates responders to immune checkpoint inhibitors

**DOI:** 10.1007/s00432-024-05628-2

**Published:** 2024-02-21

**Authors:** Ludmila Krizova, Iva Benesova, Petra Zemanova, Jan Spacek, Zuzana Strizova, Zuzana Humlova, Veronika Mikulova, Lubos Petruzelka, Michal Vocka

**Affiliations:** 1 Department of Oncology, General University Hospital in Prague and First Faculty of Medicine, Charles University, U Nemocnice 499/2, 128 00 Prague 2, Czech Republic; 2 Department of Immunology, Second Faculty of Medicine, Charles University in Prague and University Hospital in Motol, Prague, Czech Republic; 3 Institute of Immunology and Microbiology, First Faculty of Medicine, Charles University, Prague, Czech Republic; 4 Institute of Medical Biochemistry and Laboratory Diagnostics, Laboratory of Clinical Immunology and Allergology, General University Hospital in Prague and First Faculty of Medicine, Charles University, Prague, Czech Republic

**Keywords:** NSCLC, Immunotherapy, Checkpoint inhibitors, Biomarkers

## Abstract

**Purpose:**

Immune checkpoint inhibitors (ICIs) dramatically changed the prognosis of patients with NSCLC. Unfortunately, a reliable predictive biomarker is still missing. Commonly used biomarkers, such as PD-L1, MSI, or TMB, are not quite accurate in predicting ICI efficacy.

**Methods:**

In this prospective observational cohort study, we investigated the predictive role of erythrocytes, thrombocytes, innate and adaptive immune cells, complement proteins (C3, C4), and cytokines from peripheral blood of 224 patients with stage III/IV NSCLC treated with ICI alone (pembrolizumab, nivolumab, and atezolizumab) or in combination (nivolumab + ipilimumab) with chemotherapy. These values were analyzed for associations with the response to the treatment and survival endpoints.

**Results:**

Higher baseline Tregs, MPV, hemoglobin, and lower monocyte levels were associated with favorable PFS and OS. Moreover, increased baseline basophils and lower levels of C3 predicted significantly improved PFS. The levels of the baseline immature granulocytes, C3, and monocytes were significantly associated with the occurrence of partial regression at the first restaging. Multiple studied parameters (*n* = 9) were related to PFS benefit at the time of first restaging as compared to baseline values. In addition, PFS nonbenefit group showed a decrease in lymphocyte count after three months of therapy. The OS benefit was associated with higher levels of lymphocytes, erythrocytes, hemoglobin, MCV, and MPV, and a lower value of NLR after three months of treatment.

**Conclusion:**

Our work suggests that parameters from peripheral venous blood may be potential biomarkers in NSCLC patients on ICI. The baseline values of Tregs, C3, monocytes, and MPV are especially recommended for further investigation.

**Supplementary Information:**

The online version contains supplementary material available at 10.1007/s00432-024-05628-2.

## Background

In the last decade, immunotherapy has considerably changed the therapeutic approach for nonsmall cell lung cancer (NSCLC). According to the current guidelines, most patients diagnosed with NSCLC receive immune checkpoint inhibitors (ICI) in monotherapy, combination therapy, or in combination with chemotherapy. Programmed death-ligand 1 (PD-L1) is an immune inhibitory transmembrane protein primarily expressed by antigen-presenting and tumor cells. Its interaction with the programmed death protein 1 (PD-1) in various immune cells, in particular activated T cells, hampers an effective antitumor immune response (especially) by initiating apoptosis of T-cells. Therefore, expression of this protein is one of the most successful tumor escape mechanisms (Pawelczyk et al. 2019). In the tumor microenvironment (TME) of NSCLC, both cancer cells and immune cells express PD-L1 (Scheel et al. 2016). It is the most common biomarker used as a stratification factor in clinical trials; its predictive value is, however, limited. Many trials showed immunotherapy benefits regardless of the degree of PD-L1 expression and not all NSCLC patients with PD-L1 expression respond to ICI (Mathew et al. 2017). Despite the large number of patients treated with ICIs, no reliable predictive biomarker for a patient’s response to ICIs is known (Lu et al. 2019). Other predictive biomarkers, such as tumor mutation burden (TMB) or microsatellite instability (MSI) cannot be used as reliable predictive biomarkers for this purpose. TMB has shown contradictory results, primarily because of different calculation methods used in individual studies (McGrail et al. 2021). MSI is, on the other hand, a relatively accurate predictor of response to ICI in several cancer types. However, as only 1% of patients with NSCLC are microsatellite unstable, its clinical value is low for this cancer type (Luchini et al. 2019).

This paucity of reliable predictors has led to further search for easily accessible and inexpensive biomarkers. Although the theoretical hypothesis that specific peripheral leukocyte levels could correlate with the response to checkpoint inhibitors has fueled multiple studies in this field, inconsistent results have been obtained (An et al. 2021).

In the presented study focusing on patients with NSCLC treated with immunotherapy, we aimed to provide comprehensive information on peripheral blood cells and their potential to serve as predictive biomarkers for treatment response and patient outcomes. Specifically, we analyzed erythrocytes, thrombocytes, both innate and adaptive immune cells (granulocytes, monocytes, and lymphocytes), and humoral immune response (namely complement compounds C3 and C4, and cytokines IFN-γ, IL-2, IL-4, IL-10, IL-12, IL-17, and TNF-α). Subsequently, we analyzed the association of each parameter with progression-free survival (PFS) and overall survival (OS). In a subgroup of these patients, we performed a second sampling 3 months after the ICI therapy initiation, at the time of their first restaging. We correlated baseline sampling with treatment results and analyzed the changes in each parameter and its association with PFS and OS.

Using this approach, we aimed to find parameters that could help identify NSCLC patients who will benefit from ICI treatment. To the best of our knowledge, this is the first study evaluating such a complex battery of peripheral blood parameters for this purpose.

## Methods

### Patients

All consecutive patients with stage III/IV NSCLC indicated for treatment with immune checkpoint inhibitors who presented at the Department of Oncology, First Faculty of Medicine Charles University, and General University Hospital in Prague from July 2018 to July 2022, agreed with inclusion in the study and signed an informed consent, were included in the study.

All patients signed an informed consent approved by the Ethics Committee of the General University Hospital in Prague. The project has followed the principles outlined in the Declaration of Helsinki for all human or animal experimental investigations.

### Data collection

The baseline peripheral venous blood was collected into EDTA tubes before the first application of ICIs and used for determining multiple peripheral blood parameters (see Supplementary Table 1). The second blood collection was made at the time of the first restaging imaging (CT or PET/CT), i.e., 3 months after the initiation of the treatment., unless the patient’s condition progressed too rapidly. The findings were evaluated according to iRECIST criteria (Seymour et al. 2017).

### Standard blood count

Standard blood count parameters were analyzed on Sysmex XN-3100™ Automated Hematology Analyzer. This instrument uses hydrodynamic focusing for erythrocytes and platelets assessment and Semiconductor-Based Flow Cytometry to assess other blood cells (Leu, Neu, Im gran, Ly, Mo, Eos, Bas).

### Cell isolation and flow cytometry

We conducted flow cytometry staining to investigate immune cell subsets in the peripheral venous blood of NSCLC patients. Initially, 50 μl of peripheral blood were used per staining panel employing the following monoclonal antibodies: CD45 KrO, CD3 PB, CD8 APC-Alexa Fluor 700, CD4 PB, HLA-DR APC, CD127 FITC, CD25 ECP, CD19 ECD, CD27 PB, IgD FITC, CD5 PC5.5, CD3 APC-Alexa Fluor 750, CD56 PE, and CD16 PE (Supplementary Tables 2, 3). Samples were briefly vortexed and incubated for 15 min in the dark at room temperature (RT). Afterward, 500 µl of VersaLyse Lysing Solution (Beckman Coulter Brea, California, USA) was added and samples were further vortexed and incubated for 20 min in the dark at RT. After that, samples were immediately analyzed by Navios EX Flow Cytometer (Backman Coulter) with subsequent analysis with Kaluza Analysis 2.1 software (Beckman Coulter). The panels were validated using the Fluorescence minus one (FMO) procedure.

### Intracellular cytokine production

The intracellular cytokine production was assessed by flow cytometry as previously described in detail (Mareckova et al. 2002). In brief, we have stimulated 500 µl of heparinized whole venous blood with cell-specific mitogens in the presence of a transport inhibitor Brefeldin A (final concentration 0.5 mg/ml; all these chemicals were obtained from Merck Life Sciences, Darmstadt, Germany). Lipopolysaccharide was used for monocytes (final concentration of 0.1 mg/ml), while for T-cells, phorbol myristate acetate (final concentration 0.1 mg/ml) and ionomycin (final concentration 0.05 mg/ml) were applied. The mixtures were incubated for 4 h at 37 °C in 5% CO_2_ in a humidified atmosphere. FACS lysis solution (BD Bioscience, San Jose, USA) was used for the erythrocytes lysis and subsequently, the samples were washed and fixed (Cells Wash, FACS permeabilizing Solution, BD Bioscience). To assess the T-cell immune responses, intracellular cytokine staining was performed with the following monoclonal antibodies: CD3-PerCP, TNF-α FITC, and IL-17 Alexa Fluor 647 procured from BD Bioscience, and IFN-γ FITC, IL-4 PE, and IL-2 APC obtained from Invitrogen Waltham, USA. Intracellular cytokine production by peripheral blood monocytes was analyzed with monoclonal antibodies CD14 FITC, IL-10 PE, IL-12, and APC (BD Bioscience). After the incubation and washing, samples were measured and analyzed on a flow cytometer BriCyte E6 (Mindray, China). T-cells were gated using forward scatter (FSC) and CD3, whereas monocytes were gated using side scatter (SSC) and CD14.

### Complement

Serum complement levels were determined using the BN™ II System (Siemens, Germany), a reliable nephelometric analyzer. The protocol established by the manufacturer was followed during the analysis.

### Statistical analysis

Graph Pad Prism 9.4.1 (Graph Pad Software Inc., USA) was used for statistical analysis. The differences between variables were evaluated using the Mann–Whitney test. PFS and OS were performed using Mantel–Haenszel statistics comparing lower and upper terciles. The receiver-operating characteristic (ROC) curve was used for the assessment of the prediction accuracy. Patients with PFS ≥ 6 months and OS ≥ 12 months were considered the benefit groups. Kaplan–Meier survival curves were used to describe differences in survival benefit, which were tested using the log-rank test. All statistical tests were performed at the level of significance of *p* < 0.05.

## Results

We have enrolled a total of 224 patients at stages III/IV NSCLC treated with checkpoint inhibitors (42 in combination with chemotherapy). Twenty-two patients were excluded from the study. The basic clinical characteristics of patients are described in Table 1. The median follow-up was 21.8 months. Control sample analysis after 3 months was performed in 174 patients. Twenty-eight patients did not undergo the second blood sampling and control CT due to the rapid progression of their disease, which prevented them from visiting our department or caused death.

**Table 1:** Characterization of the studied population for whom complete clinicopathological data were available for subsequent analyses

	*n* = 202 *n* (%)
Mean age at the time of initiation of immunotherapy (years)	70.1 (37.9–85.9)
Sex	
Female	91 (45.0)
Male	111 (55.0)
Histology	
Adenocarcinoma, NOS	133 (65.8)
Squamous cell carcinoma	57 (28.2)
Large cell carcinoma	6 (3.0)
Adenosquamous carcinoma	6 (3.0)
Extent of the disease	
Locally advanced	18 (8.9)
Metastatic	184 (91.2)
Localization of metastases	
Bone	56 (30.4)
Lung	101 (54.9)
Brain	11 (6.0)
Liver	34 (18.5)
Adrenal gland	41 (22.3)
Nonregional lymphatic nodules	39 (21.2)
Other	22 (12.0)
Immunotherapy type	
Pembrolizumab mono	91 (45.1)
Nivolumab mono	93 (46.0)
Ipilimumab + nivolumab	13 (6.4)
Atezolizumab mono	5 (2.5)
Line of treatment	
First line	111 (55.0%)
Second line	71 (35.2%)
Third and higher line	20 (9.9%)

### Association of baseline parameters with PFS and OS

To evaluate the association of individual parameters with the efficacy of ICI, we divided patients into two groups according to the effects of ICI treatment on PFS and OS. The PFS benefit was defined as PFS > 6 months, while patients with PFS < 6 months constituted the nonbenefit group. The OS benefit group consisted of patients with OS of > 12 months (patients with OS < 12 months constituted the OS nonbenefit group).

First, we compared the associations between the baseline peripheral blood parameters and the tested survival endpoints (PFS, OS) in the benefit and nonbenefit groups in whole tested population (Table 2). Better PFS was associated with lower baseline levels of immature granulocytes, monocytes, C3, and with higher levels of basophils, Tregs, hemoglobin, and mean platelet volume (MPV). Similarly, better OS was found in patients with lower baseline levels of monocytes and higher levels of Tregs, TNF-α, hemoglobin, and MPV. No significant differences were found in the other studied parameters (Supplementary Table 4).

**Table 2:**
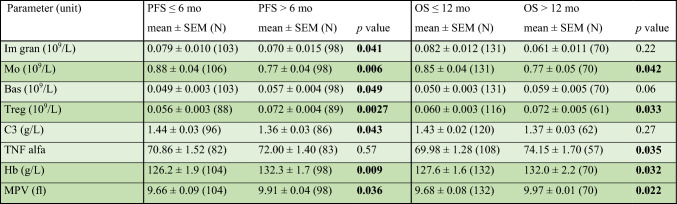
Association of baseline parameters with PFS and OS

The comparison of each evaluated parameter from the perspective of PFS (≤ 6 vs > 6 mo) and OS (≤ 12 vs > 12 mo) was made using the Mann–Whitney test. Only parameters with at least one statistically significant result are reported in this table

*Bas* basophils, *Hb* hemoglobin, *Im gran* immature granulocytes, *Mo* monocytes, *MPV* mean platelet volume, *OS* overall survival, *PFS* progression-free survival, *SEM* standard error of the mean, *TNF alfa* tumor necrosis factor alfa, *Treg* regulatory T cells

**Fig. 1:**
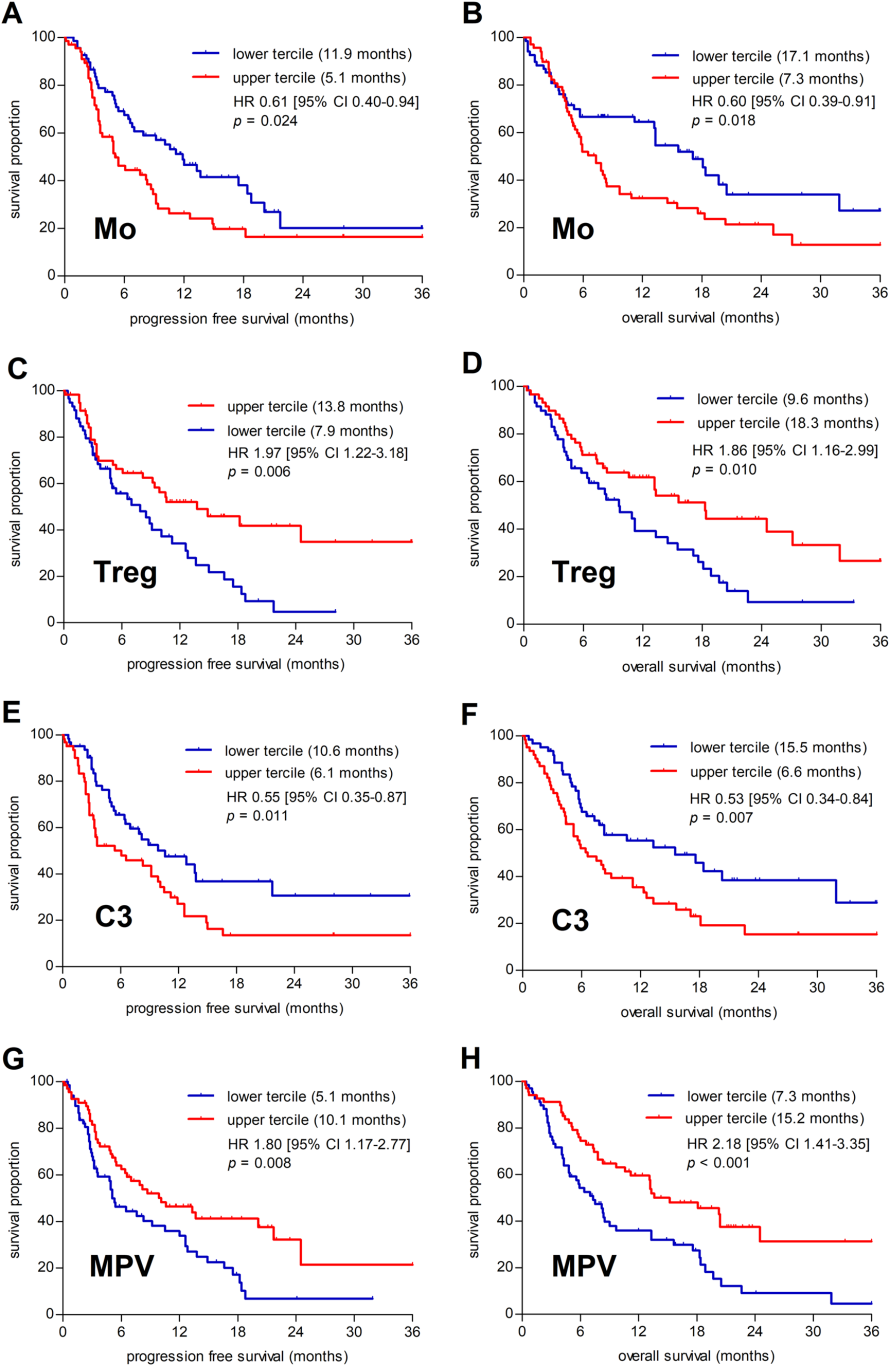
Kaplan–Meier curves for the lower and upper terciles of Mo, Treg, C3 and MPV; median survival for upper and lower terciles of the respective parameters are indicated in the legends of individual graphs. **A** Kaplan–Meier progression-free survival (PFS) curve of the lower and upper terciles of the baseline monocyte level. **B** Kaplan–Meier survival (OS) curve of the lower and upper terciles of the baseline monocyte level. **C** Kaplan–Meier progression-free survival (PFS) curve of the lower and upper terciles of the baseline Treg level. **D** Kaplan–Meier survival (OS) curve of the lower and upper terciles of the baseline Treg level. **E** Kaplan–Meier progression-free survival (PFS) curve of the lower and upper terciles of the baseline C3 level. **F** Kaplan–Meier survival (OS) curve of the lower and upper terciles of the baseline C3 level. **G** Kaplan–Meier progression-free survival (PFS) curve of the lower and upper terciles of the baseline MPV level. **H** Kaplan–Meier survival (OS) curve of the lower and upper terciles of the baseline MPV level

Subsequent survival analyses comparing the lower and upper terciles of each baseline parameter confirmed the previous results. Lower levels of monocytes, C3, and MPV and higher levels of Tregs were associated with better PFS and OS (Fig. 1)**.** Patients with higher baseline levels of CD3^+^ T cells, eosinophils, and hemoglobin showed a better PFS but not OS. Increased levels of CD19^+^ B cells and basophils were associated with better OS, as opposed to high levels of immature granulocytes and IL-4 that were associated with worse OS (Supplementary Table 5).

Next, we compared the levels of baseline peripheral blood parameters among patients with complete response (CR)/partial response (PR), stable disease (SD), and progressive disease (PD). As expected, patients with CR/PR on first restaging had better PFS and OS compared to those with SD or PD (Supplementary Fig. S1). Initial lower levels of immature granulocytes (*p* = 0.008), C3 (*p* = 0.035), and monocytes (*p* = 0.019) were significantly associated with CR/PR at the first restaging (Table 3); no significant association was found in the other evaluated parameters (Supplementary Table 6).

**Table 3:**
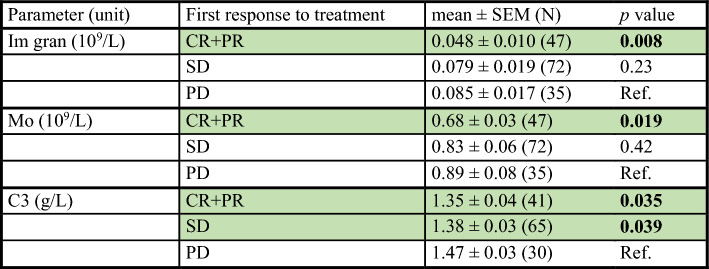
Association of baseline parameters with the initial response to ICI treatment

The comparison of each evaluated parameter according to the first response to the ICI treatment (CR + PR vs PD, SD vs PD) was made using the Mann–Whitney test. Only parameters with at least one statistically significant result are reported in this table

*SEM* standard error of the mean, *CR* complete response, *PR* partial response, *SD* stable disease, *PD* progressive disease, *Im gran* immature granulocytes, *Mo* monocytes

**Fig. 2:**
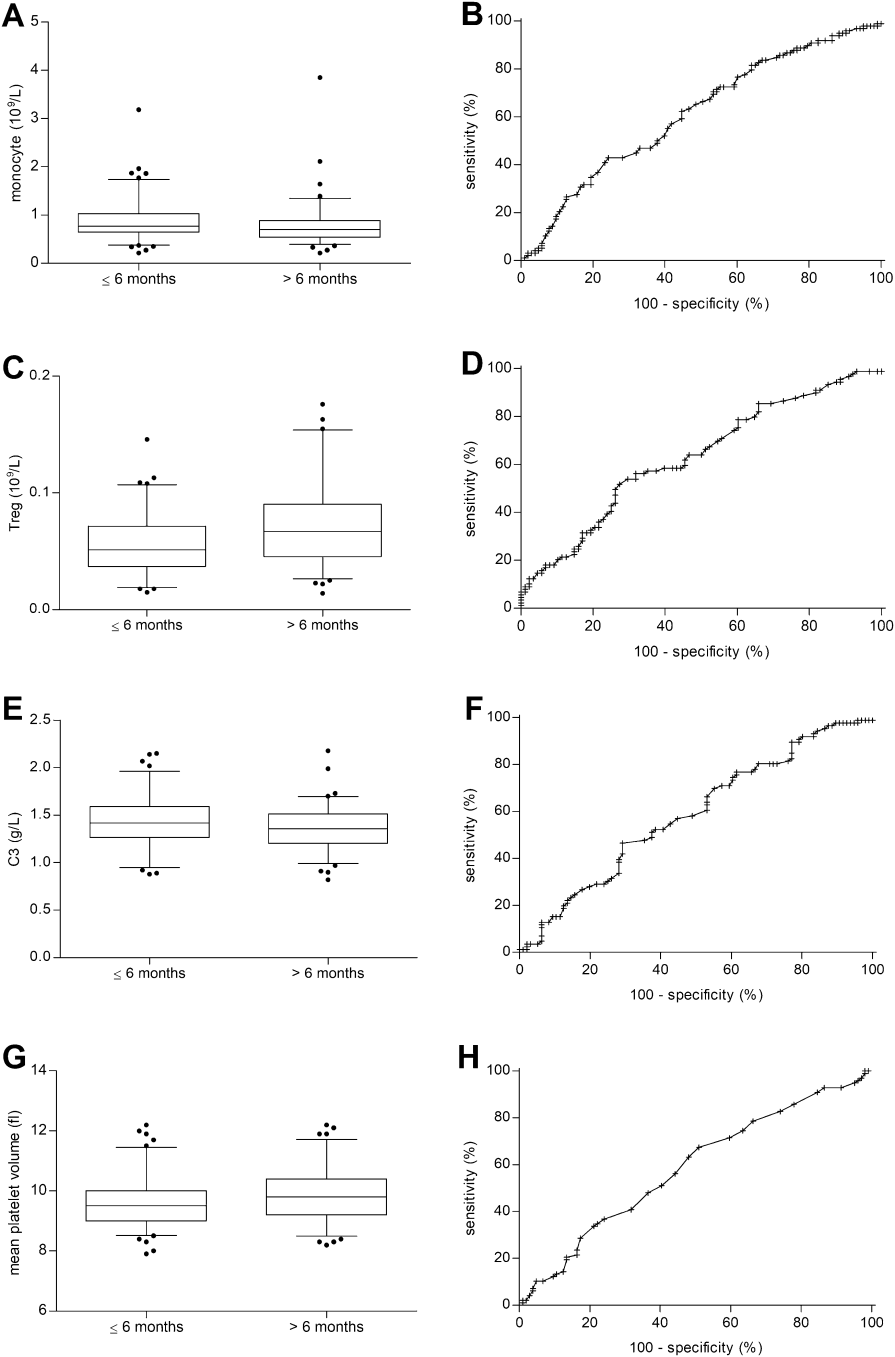
Analysis of potential predictive biomarkers. **A** Differences of monocytes between the PFS benefit and nonbenefit groups. **B** ROC curve of monocytes. **C** Differences of Treg between the PFS benefit and nonbenefit groups. **D** ROC curve of Treg. **E** Differences of C3 between the PFS benefit and nonbenefit groups. **F** ROC curve of C3. **G** Differences of mean platelet volume between PFS benefit and nonbenefit groups. **H** ROC curve of mean platelet volume

Based on the above-described results, we investigated the potential predictive value of four selected biomarkers (baseline level of monocytes, Tregs, C3, and MPV). We have to conclude that neither the baseline level of monocytes, Tregs, C3, or MPV are useful as predictive biomarkers of PFS prolongation in patients treated with ICIs (area under the curve [AUC] of the ROC for monocytes 0.612, Treg 0.631, C3 0.587 and MPV 0.585) (Fig. 2). Similar results were observed for the neutrophil-to-lymphocyte ratio (NLR) with AUC 0.541.

We further focused on the significance of NLR as a nonlinear biomarker and analyzed the difference in PSF and OS in patients with low versus high baseline NLR (threshold was set to 5). Statistically significantly better survival (PFS and OS) was observed in patients with NLR ≤ 5 (Supplementary Fig. S2A and B). Baseline level of eosinophils higher than 0.13 10^9^/L was also associated with better PSF, but not with improved OS (Supplementary Fig. S2C and D). **T**o reduce some heterogenity in the data and potential bias caused by different types of therapy administered, we performed a subgroup analysis of patients treated with PD-1 inhibitors only (the small number of patients treated with other agents or their combination did not allow further subgroup analysis). The PFS analysis was consistent with the analysis of the overall study population. Better PFS was associated with baseline levels of immature granulocytes, monocytes, C3, basophils, Tregs, hemoglobin, and mean platelet volume (MPV). Better OS in the population treated with PD-1 inhibitors was observed in patients with lower baseline levels of monocytes and higher levels of hemoglobin and MPV. (Supplementary Tables 7 and 8).

### Analysis of selected parameters after 3 months of ICI therapy

In 174 patients, the blood count parameters were evaluated also at the time of the first restaging (after three months of the treatment, Table 4). We observed a significant association between the PFS benefit and higher levels of lymphocytes, basophils, erythrocytes, hemoglobin, MCV, and MCH as well as lower levels of neutrophils, immature granulocytes, and NLR at the follow-up treatment. The higher values of lymphocytes, erythrocytes, hemoglobin, MCV, and MPV, as well as lower values of NLR, were associated with statistically significantly longer OS.

**Table 4:**
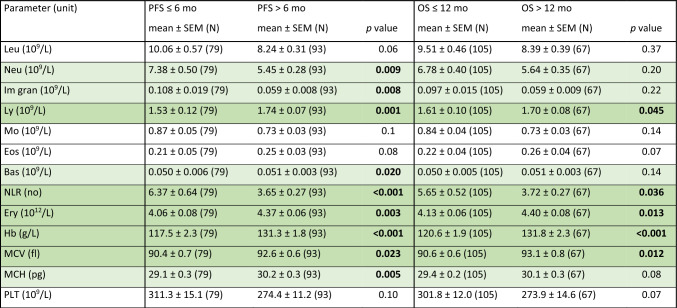
Association between parameters after 3 months of therapy with PFS and OS

Comparison of each parameter according to PFS (≤ 6 vs > 6 mo) and OS (≤ 12 vs > 12 mo) using the Mann–Whitney test after 3 months of therapy

*Bas* basophils, *Eos* eosinophils, *Ery* erythrocytes, *Hb* hemoglobin, *Im gran* immature granulocytes, *Leu* leukocytes, *Ly* lymphocytes, *Mo* monocytes, *MCV* mean corpuscular volume, *MCH* mean cell hemoglobin, *MPV* mean platelet volume, *Neu* neutrophils, *NLR* neutrophils-to-lymphocytes ratio, *OS* overall survival, *PLT* platelets, *PFS* progression-free survival, *SEM* standard error of the mean

Subsequent analysis of the differences in individual parameters between the individual time points within the benefit/nonbenefit groups revealed a significant decrease in hemoglobin in both nonbenefit groups (*p* = 0.005 for PFS and *p* = 0.015 for OS, respectively). Besides, the lower levels of lymphocytes after 3 months of treatment were observed in the PFS nonbenefit group. Interestingly, neutrophils and NLR counts were also reduced in PFS and OS benefit groups (Supplementary Table 9).

In addition, we calculated the mean change of each parameter after three months of therapy in individual patients (Table 5). A significant increase in the levels of neutrophils was observed in both nonbenefit groups while in both benefit groups, a significant decrease was recorded. A similar observation was made for NLR. The mean change in the lymphocyte levels was positive in the PFS benefit group, unlike in the nonbenefit group; this difference was also statistically significant. From nonimmune parameters, the change in MCH between time points was negative in the PFS nonbenefit group but positive in the PFS benefit group. The decreases in MCV and levels of hemoglobin were much more pronounced in both nonbenefit groups. Where erythrocytes are concerned, the same observation was valid for OS.

**Table 5:**
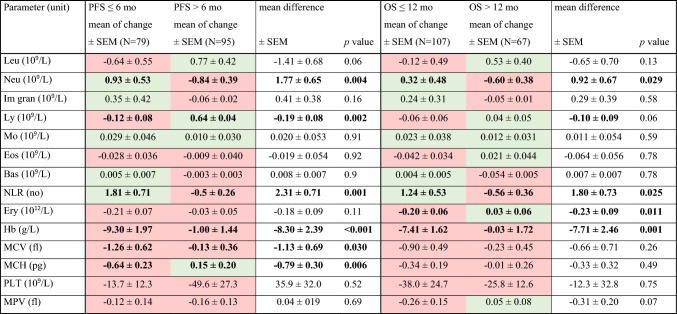
Association between the mean change in each parameter during therapy with PFS and OS (decrease is highlighted in red, increase in green; statistically significant differences are in bold)

Comparison of mean changes in each parameter classified according to PFS (≤ 6 vs > 6 mo) and OS (≤ 12 vs > 12 mo) using the Mann–Whitney test after 3 months of therapy

*Bas* basophils, *Eos* eosinophils, *Ery* erythrocytes, *Hb* hemoglobin, *Im gran* immature granulocytes, *Leu* leukocytes, *Ly* lymphocytes, *Mo* monocytes, *MCV* mean corpuscular volume, *MCH* mean cell hemoglobin, *MPV* mean platelet volume, *Neu* neutrophils, *NLR* neutrophils-to-lymphocytes ratio, *OS* overall survival, *PLT* platelets, *PFS* progression-free survival, *SEM* standard error of the mean

## Discussion

Despite the widespread use of ICI therapy in oncology, current predictive biomarkers are unreliable. There is, therefore, an urgent clinical need to identify markers that can predict patients who are likely to have a good therapeutic response to ICI to decide whether to treat them with immunotherapy alone or in combination with short chemotherapy. Commonly used markers, such as PD-L1, TMB, and MSI are not sufficiently accurate in predicting long-term survival in patients treated with ICI. A combination of biomarkers could be a more effective approach (Sankar et al. 2022). Similarly, patients with a poor therapeutic response to ICI must be identified, as they will be candidates for new therapeutic approaches combining other modalities. Therefore, we focused on easily accessible cells in the peripheral blood and their association with the response to the ICI therapy and the overall survival of each patient. ICIs are typically used in nearly all patients with NSCLC as the first- or second-line treatment and gradually move into curative (adjuvant and neoadjuvant) settings, where the need for accurate prediction of the benefit to patients is even more important (Ettinger et al. 2023). Predictive biomarkers from peripheral blood could help resolve the frequent problem of the availability (and representativeness) of the biopsied tissue for molecular analysis as repeated biopsies burden the patient.

### Leukocytes

Currently approved immunotherapeutic approaches for the treatment of solid cancers focus mainly on T-lymphocytes (Johnson et al. 2022). The therapy aims to restore their anticancer properties by blocking various immunosuppressive mechanisms, such as PD-1/PD-L1, CTLA-4/CD80-CD86, and LAG-3/MHC II interactions (Chocarro et al. 2021; Wei et al. 2018). The role of B lymphocytes in ICI therapy is less explored, even though they also express CD80/CD86, PD-1, and PD-L1, which are direct targets of ICIs or are affected by ICIs (Kim et al. 2021; Patel et al. 2020). B-lymphocytes abundance within the TME, especially within the tertiary lymphoid structures, correlates with the response to ICI therapy in various human cancers, including NSCLC (Kim et al. 2021). Consistent with these observations, we showed that higher levels of CD19^+^ B-cells at the baseline are associated with better OS in patients treated with ICI.

Owing to the important role of both lymphocyte populations, it has been hypothesized that the lymphocyte count in peripheral blood correlates with the efficacy of ICI treatment. In a retrospective study on 231 patients with NSCLC who were treated with anti-PD1 immunotherapy (nivolumab, pembrolizumab and atezolizumab), higher peripheral lymphocyte counts observed prior to and one month after the start of ICI treatment were correlated with better PSF and OS (Lee et al. 2022). Previous studies have demonstrated an association between better outcomes of ICI and an increase in the levels of CD4^+^ T cells, NK cells, and Tregs and a decrease in levels of CD8^+^ T cells in the peripheral blood. In the cohort treated with chemotherapy without ICI, the opposite trend was observed in CD4^+^ T cells, CD8^+^ T cells, Tregs, and B cells (Yan et al. 2022).

In our study, the baseline peripheral lymphocyte count was not associated with a response to the ICI therapy, even in the anti-PD1 therapy only subgroup; however, higher lymphocyte counts at the time of first restaging was associated with better PFS and OS. Moreover, lymphocyte counts after three months of treatment were significantly decreased in patients in the PFS nonbenefit group. The vast diversity of lymphocytes, with roles ranging from highly effective antitumor lymphocytes to regulatory T- and B-cells, presents a challenge for the detection of associations. We, therefore, focused on the individual subtypes of lymphocytes in the peripheral blood, especially T-lymphocytes.

In our study, better PFS was detected in patients with higher baseline levels (upper tercile) of CD3^+^ T-cells. In addition, increased CD4^+^ T-cell count tended (albeit insignificantly, *p* = 0.09) to improve PFS. Physiologically, Tregs maintain airway tolerance, a type of immunological surveillance; in lung cancer, however, they suppress antitumor immunity through various mechanisms. Therefore, Treg abundance in the peripheral blood and tumor tissues is associated with poor prognosis in NSCLC patients (Principe et al. 2021). Owing to the expression of the immunotherapeutic targets PD-L1 and CTLA-4 on the surface of Tregs, patients with increased levels of Tregs could be ideal candidates for ICIs therapy. Higher baseline levels of Tregs were associated with better treatment prognosis in our cohort of patients with NSCLC, which is in accordance with observations from several previous studies (Yan et al. 2022; Koh et al. 2020). However, Kagamu et al. reported the opposite effect of baseline levels of Tregs (Kagamu et al. 2020). Notably, the outcome of ICI therapy probably depends on the Tregs phenotype. Kumagai et al. described a poor response in NSCLC patients with high levels of tumor-infiltrating PD-1^+^ Tregs (Kumagai et al. 2020).

None of the other lymphocyte subtypes were significantly associated with ICI efficacy or patient prognosis. The variability of designs among studies makes the results difficult to compare and prospective trials are needed to address this issue. Based on our cohort, and considering other results, we suggest focusing on Tregs in future studies.

Myeloid-derived suppressor cells (MDSCs), an important part of innate immunity, are a heterogenous population of immunosuppressive cells. These cells limit antitumor responses in the TME by inhibiting effector T and NK cells, inducing an immunosuppressive environment, and promoting angiogenesis. They probably contribute to ICI treatment outcomes (Yang et al. 2020). Although most studies have examined their effect on the TME, recent studies have analyzed their presence in the peripheral blood and their relation to on the ICI response (Peranzoni et al. 2020). Lower MDSC levels were associated with better ICI treatment outcomes (Parikh et al. 2018). We did not analyze MDSCs; we only measured the peripheral count of monocytes. We observed a significant association between lower baseline monocyte levels and better survival (both PFS and OS). Lower baseline monocyte levels were significantly associated also with a better response to immunotherapy (CR/PR), as assessed at the first restaging. In contrast with our study, Parikh et al. found no association between baseline monocyte count and PFS/OS in their cohort of 32 patients with NSCLC (Parikh et al. 2018). However, this might be caused by the low number of patients in their study, and, therefore, its insufficient power. Khunger et al. reported similar results to Parikh et al. for levels of baseline or post-treatment monocyte counts in 109 patients with NSCLC (Khunger et al. 2018).

Eosinophils and their potential as predictive biomarkers of response to ICI and immune-related side effects are an emerging topic in immuno-oncology research. So far, few studies have focused on this; however, eosinophils seem to play a role in antitumor immunity (Grisaru-Tal et al. 2022). Although their granules contain cytotoxic proteins, eosinophils may play a role in immunosuppression (Treg recruitment, M2 polarisation, IDO expression, etc.) and support tumor cells through the production of various growth factors. Higher baseline eosinophils (the threshold is commonly set between 0.125 and 0.135 × 10^9^/L) were previously associated with better outcomes in NSCLC patients treated with immunotherapy (Sibille et al. 2022). In 158 NSCLC patients, high baseline eosinophil levels (≥ 0.130 × 10^9^/L) showed predictive value for the response to ICI and the occurrence of immune-related adverse events (irAEs) (Caliman et al. 2022). A study on 168 patients with NSCLC, renal cancer, or malignant melanoma reported the threshold of absolute eosinophil count to be 0.130 × 10^9^/L. Patients with higher levels of eosinophil count had a significantly higher risk of developing irAE; however, they had a longer-lasting clinical response to ICI (Giommoni et al. 2021). Our data confirmed that patients with baseline eosinophil counts higher than 0.130 × 10^9^/L had longer PFS, but no significant association with OS was detected.

Basophils represent less than 1% of peripheral blood leukocytes. The role of basophils in cancer remains unknown and their role is assumed to vary among different cancer types (Chauhan et al. 2022). They produce angiogenic factors, thus supporting tumorigenesis; on the other hand, they also release extracellular DNA traps and produce granzyme B and histamine (Marone et al. 2020). Notably, we observed that higher baseline basophil levels were correlated with longer PFS. However, to the best of our knowledge, there are no other studies investigating this issue, which calls for further research in this field. As far as we know, the only study investigating basophil counts in association with cancer prognosis was performed by Wu et al. who demonstrated that in patients with gastric cancer treated with ICI with chemotherapy, increased basophil counts were associated with poor prognosis. In controlled arm treated with chemotherapy alone peripheral basophiles had no significant association with response or survival. In this work the authors hypothesis that high peripheral basophils level might induce infiltration of tumor microenvironment by M2 macrophages. (Wu et al. 2022). Data for NSCLC are not available and further analysis containg tumor microenvirometn is needed.

Neutrophils, the most abundant myeloid cells in human blood, may support tumor cell proliferation and genetic instability and induce angiogenesis, immunosuppression, and tumor-promoting inflammation (Tazzyman et al. 2009; Oberg et al. 2019). On the other hand, they can also release nitric oxide (NO), initiate trogoptosis, and support other anticancer immune cells. Neutrophils have been reported to be the most abundant immune cells in the TME of NSCLC patients and to be negatively correlated with CD8^+^ T cells (Kargl et al. 2017). In our study, lower levels of neutrophils at the baseline and after three months of treatment were associated with better PFS. The neutrophil-to-lymphocyte ratio (NLR) has been recently given much attention in the field of immuno-oncology. Its predictive value has been studied in multiple cancer types treated with ICI. However, implementation in clinical practice remains limited, especially due to the lack of prospective data and reliable thresholds. In a meta-analysis of 17 studies including more than 2,000 patients with advanced NSCLC, baseline NLR and changes in this parameter during treatment were associated with disease outcome. In this meta-analysis, the NLR threshold of 6 was found to be optimal (Li et al. 2020). However, many studies on NSCLC used a cut-off of 5, which is generally accepted and used across the cancer types; it was used as a threshold also in our study (Derman et al. 2017; Bagley et al. 2017). Consistently with the results of the previous studies, we also found significantly longer PFS and OS in patients with a baseline NLR < 5. In the nonbenefit PFS and OS groups, a significant increase in NLR was observed. Our data confirmed the utility of NLR as a potential biomarker in immuno-oncology; however, large prospective trials should be conducted to determine the exact role of NLR in NSCLC.

### Complement

The role of the complement in antitumor immunity and ICI efficacy has not been fully elucidated to date. Although complement is largely effective against bacterial infections, its role in anticancer immunity is different. Complement receptors are present on most cell types within the TME, affecting a wide variety of cells (Roumenina et al. 2019). The complement may support carcinogenesis and immunosuppression and tumor cells can produce complement regulators to escape the immune system (Roumenina et al. 2019; Pio et al. 2019; Thurman et al. 2020). In vitro cancer cell lines and mice models showed that the C3 protein suppresses antitumor immunity by modulating tumor-associated macrophages (Zha et al. 2019). In our study, we have observed the association of lower levels of the C3 complement protein in the peripheral blood with longer PFS. To the best of our knowledge, this phenomenon has not been described so far. We hypothesize that these findings can be attributed to the immunosuppressive ability of C3.

### Platelets and MPV

We have not found any significant relationship between the platelet count and the efficacy of treatment with ICIs in NSCLC in our cohort. We, however, observed a significant association between the higher baseline mean platelet volume and longer PSF and OS. The link between MPV and prognosis of NSCLC has previously been described in multiple retrospective studies. For instance, a study of 496 NSCLC patients at stages IIIB/IV showed that a high level of MPV is associated with shorter OS (Omar et al. 2018). On the other hand, a recent meta-analysis of 2,421 patients with NSCLC from 11 publications revealed no significant associations of MPV with OS and PFS (Kharel et al. 2022). It must be, however, noted that none of the included studies was prospective, and that the meta-analysis did not specify differences between different types of treatment. Therefore, the prognostic value of MPV for response to ICI is yet to be determined. However, based on our results, it appears that MPV could be a promising parameter for this purpose.

### Hemoglobin

Higher baseline hemoglobin in NSCLC patients treated with immunotherapy is associated with better outcome. This was described on 310 patients with this diagnosis were pretreatment Hb count ≥ 110 g/L served as a favorable prognostic marker. (Zhang et al. 2020) Similar conclusion using different cut-off for hemoglobin was observed on study containding 249 ICI treated NSCLC patients. In this work authors analysed OS and RR in correlation with hemoglobin < 120 g/L or < 100 g/L. The higher baseline hemoglobin was significantly associated with better response rate and survival using both cutoffs. (Ayers et al. 2021). The result from our cohort is consistent with these data, as high baseline hemoglobin was associated with longer PFS and OS. We also observed a significant difference in the change of hemoglobin levels during the treatment between the benefiting and nonbenefiting groups of patients. A decrease in hemoglobin levels was observed in all groups, but in the nonbenefit groups, the decline was sharper.

Our work brings new data to the field of stable immunotherapy biomarkers. The analysis of an extensive set of peripheral blood parameters in a large cohort of patients treated with immunotherapy for NSCLC as well as the repeated sampling after 3 months are indisputable advantages of this study.

A potential bias of out work arisen from the analysis of patients who were treated with different checkpoint inhibitors cannot be excluded. For the purposes of our research, we considered immunotherapy as one therapeutic approach regardless of the drug used. We do not expect fundamental differences between the individual drugs because the basic mechanism of action of the checkpoint inhibitors is the same. We address this issue by performing a subgroup analysis where only patiens treated with PD-1 inhibitors were analyzed. We observed that the benefit in PFS was associated with the same baseline parameters as in the whole studied population. The OS benefit in this subgroup was associated with only three baseline parameters (monocytes, hemoglobin and MPV) compared to the whole population where five parameters were associated with better outcome. These differences may be due to the analysis of small numbers of data in the subgroup.

## Conclusion

In conclusion, our study demonstrated that even parameters from basic peripheral blood count are associated with the response to ICIs. The analysis of the components of the immune system in peripheral blood showed their association with the outcomes of ICIs treatment in NSCLC. According to our results, Tregs, monocytes, C3, and MPV seem to have prognostic value, which requires further investigation and clarification of the relationships between these parameters and the response to ICIs treatment. We also confirmed the prognostic value of NLR (using a threshold of ≤ 5) and eosinophils (using a threshold of ≤ 0.130 10^9^/l).

## Supplementary Information

Below is the link to the electronic supplementary material.

Supplementary file1 (DOCX 428 KB)

Supplementary file2 (DOCX 91 KB)

## Data Availability

Data are available on reasonable request to the corresponding author Vocka Michal (michal.vocka@vnf.cz).

## Abbreviations

Bas: Basophils
CR: Complete response
Eos: Eosinophils
Ery: Erythrocytes
FMO: Fluorescence minus one
Hb: Hemoglobin
ICIs: Immune checkpoint inhibitors
Im gran: Immature granulocytes
Leu: Leukocytes
Ly: Lymphocytes
MCH: Mean cell hemoglobin
MCV: Mean corpuscular volume
MDSC: Myeloid-derived suppressor cells
Mo: Monocytes
MPV: Mean platelet volume
Neu: Neutrophils
NLR: Neutrophile-to-lymphocyte ratio
NO: Nitric oxide
NSCLC: Nonsmall cell lung cancer
OS: Overall survival
PD-1: Programmed death protein 1
PD-L1: Programmed death-ligand 1
PFS: Progression-free survival
PD: Progressive disease
PLT: Platelets
PR: Partial response
SD: Stable disease
TME: Tumor microenvironment
TNF alfa: Tumor necrosis factor alfa
Treg: Regulatory T cells
