# Supplementary material for: Immunophenotyping of peripheral blood in NSCLC patients discriminates responders to immune checkpoint inhibitors
Source: J Cancer Res Clin Oncol. 2024 Feb 21;150(2):99. doi: 10.1007/s00432-024-05628-2 (PMC10881622; doi:10.1007/s00432-024-05628-2)

**Supplementary Figures**

**Figure S1:** Association of the initial response to ICI with PFS and OS.

**Figure S2:** Association of neutrophil-to-lymfocyte ratio (NLR) and baseline eosinophiles with progression free survival (PSF) and overall survival (OS) using Kaplan–Meier analysis.

**Figure S1** – **Association of the initial response to ICI with PFS and OS.** (A) Kaplan–Meier progression free survival (PFS) curves for different initial responses to treatment; (B) Kaplan–Meier overall survival (OS) curves for different first response to treatment.


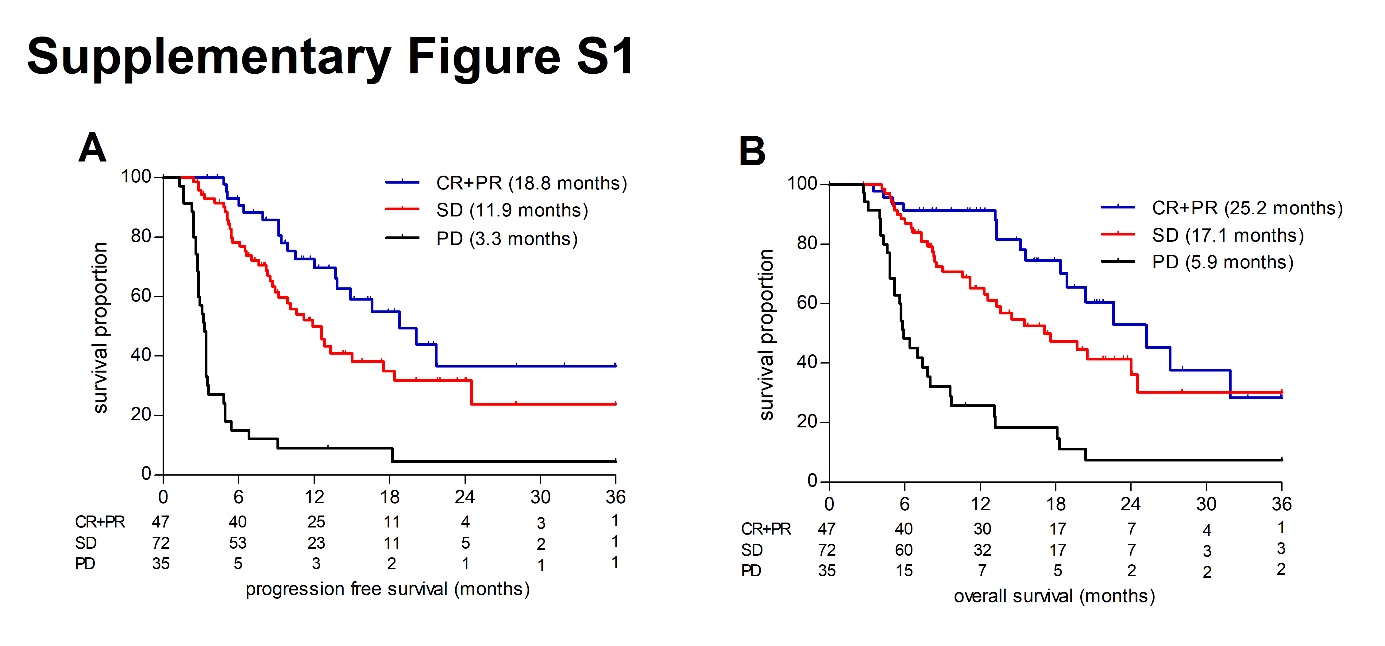


CR – complete response, PR – partial response, SD – stable disease, PD – progressive disease

**Figure S2** – **Association of neutrophil-to-lymfocyte ratio (NLR) and baseline eosinophiles with progression free survival (PSF) and overall survival (OS) using Kaplan–Meier analysis.**

(A) PFS of NLR ≤/> 5 (B) OS of NLR ≤/> 5 (C) PFS of eosinophils ≤/> 0.13 10^9^/L (D) OS of eosinophils ≤/> 0.13 10^9^/L


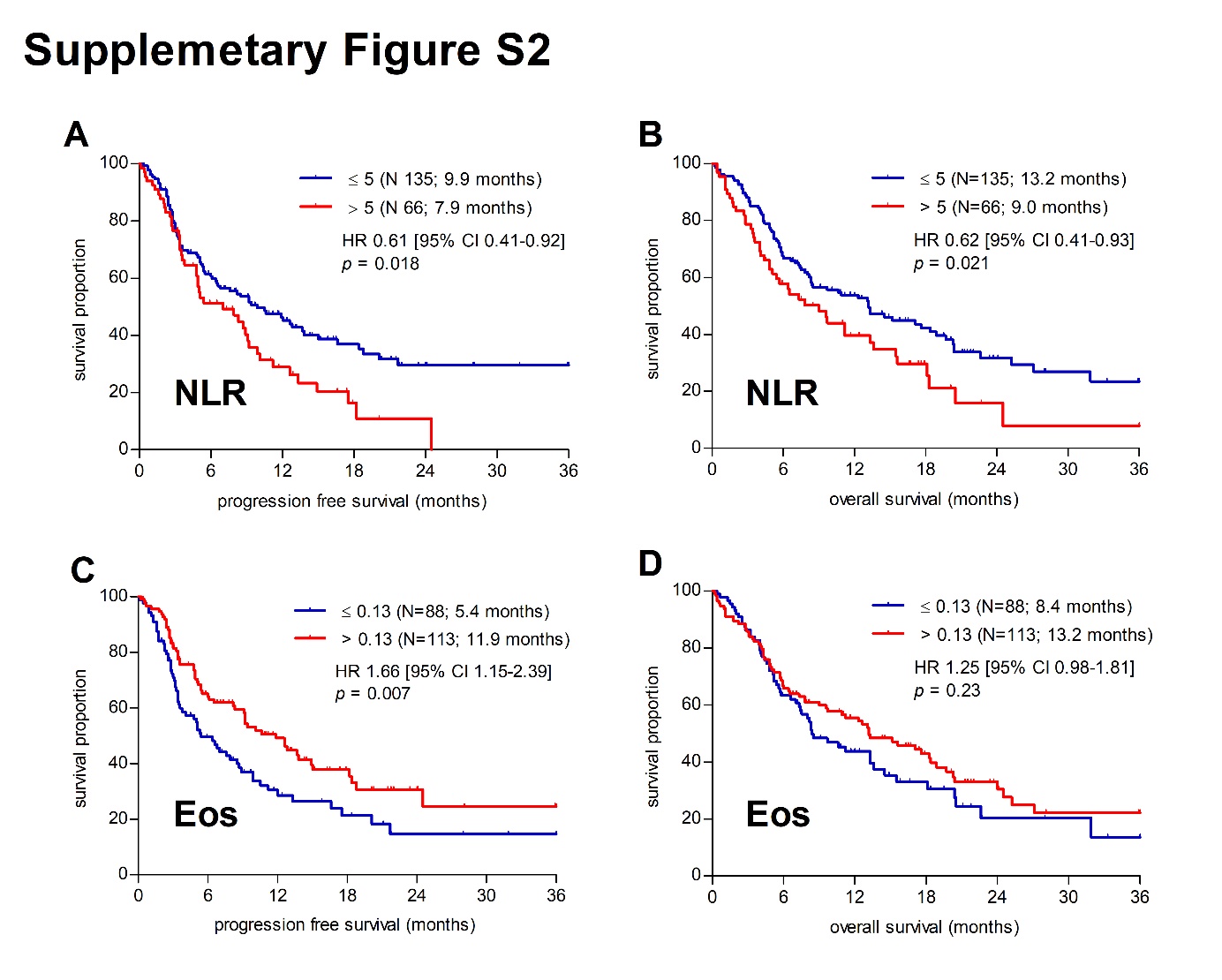

Supplement: Supplementary file 1 — Supplementary file1 (DOCX 428 KB) [file 432_2024_5628_MOESM1_ESM.docx]
