# Supplementary material for: Immunophenotyping of peripheral blood in NSCLC patients discriminates responders to immune checkpoint inhibitors
Source: J Cancer Res Clin Oncol. 2024 Feb 21;150(2):99. doi: 10.1007/s00432-024-05628-2 (PMC10881622; doi:10.1007/s00432-024-05628-2)
Supplement: Supplementary file 2 — Supplementary file2 (DOCX 91 KB) [file 432_2024_5628_MOESM2_ESM.docx]

**Supplementary Tables**

**Table S1:** Investigated parameters.

**Table S2:** Flow cytometry antibodies used for immunophenotyping.

**Table S3:** Flow cytometry antibodies used for intracelular staining of cytokines production.

**Table S4:** Comparison of individual parameters in groups classified according to PFS (≤6 vs >6 mo) and OS (≤12 vs >12 mo) using Mann-Whitney test.

**Table S5:** Analysis of PFS and OS using Mantel-Haenszel statistics comparing lower and upper terciles of each category.

**Table S6:** Comparison of individual parameters in groups classified according to the initial response to therapy (CR+PR vs PD, SD vs PD) using Mann-Whitney test.

**Table S7:** Comparison of individual parameters patient treated with anti-PD1 only in groups classified according to PFS (≤6 vs >6 mo) using Mann-Whitney test.

**Table S8:** Comparison of individual parameters patient treated with anti-PD1 only in groups classified according to OS (≤12 vs >12 mo) using Mann-Whitney test.

**Table S9:** Comparison of the individual parameters before and after therapy according to PFS (≤6 vs >6 mo) and OS (≤12 vs >12 mo) using Mann-Whitney test.

**Table S1**: Investigated parameters.

| Cellular immunity | CD45+ leukocytes, monocytes, basophils, eosinophils, neutrophils, immature granulocytes, CD3-CD56+CD16+ NK cells, CD3+CD56+CD16+ NKT cells, CD3+ T cells, CD3+HLA-DR+ activated T cells CD3+CD4+ T cells, CD4+CD45RA+ T cells, CD3+CD4+*CD25+CD127dim* regulatory T cells, CD3+CD8+ T cells, CD19+ B cells, CD19+CD5+ B cells, neutrophils to lymphocytes ratio (NLR) | |
| --- | --- | --- |
| Humoral immunity | Complement | C3, C4 |
|  | Cytokines | IFN-γ, IL-2, IL-4, IL-10, IL-12, IL-17, TNF-α |
|  | Immunoglobulins | IgA,IgE, IgG1-4, IgM |
| Other parameters | Erythrocytes, hemoglobin, MCV, MCH, thrombocytes, MPV | |

**Table S2:** Flow cytometry antibodies used for immunophenotyping.

| **Panel** | **Target** | **Fluorophore** | **Manufacturer** | **Clone** | **Volume per test (µL)** |
| --- | --- | --- | --- | --- | --- |
| T-cells | CD3 | PB | Beckman Coulter | UCHT1 | 2.5 |
|  | CD8 | APC-Alexa Fluor 700 | Beckman Coulter | B9.11 | 2.5 |
|  | CD4 | PB | Beckman Coulter | 13B8.2 | 2.5 |
|  | HLA-DR | APC | Beckman Coulter | Immu-357 | 5.0 |
| T-regs | CD4 | PB | Beckman Coulter | 13B8.2 | 2.5 |
|  | CD127 | FITC | Beckman Coulter | R34.34 | 2.5 |
|  | CD25 | ECD | Beckman Coulter | B1.49.9 | 5.0 |
| B-cells | CD19 | ECD | Beckman Coulter | J3-119 | 2.5 |
|  | CD27 | PB | Beckman Coulter | 1A4CD27 | 5.0 |
|  | IgD | FITC | Beckman Coulter | IA6-2 | 5.0 |
|  | CD5 | PE-Cy5.5 | Beckman Coulter | BL1A | 2.5 |
| NK/NKT cells | CD3 | APC-Alexa Fluor 750 | Beckman Coulter | UCHT1 | 2.5 |
|  | CD56 | PE | Beckman Coulter | N901 (NKH-1) | 2.5 |
|  | CD16 | PE | Beckman Coulter | 3G8 | 2.5 |
| Leukocytes | CD45 | Krome Orange | Beckman Coulter | J33 | 2.5 |

**Table S3:** Flow cytometry antibodies used for intracelular staining of cytokines production.

| Panel | Target | Fluorophore | Manufacturer | Clone | Volume per test (uL) |
| --- | --- | --- | --- | --- | --- |
| Lymphocytes | CD3 | PerCP | Becton Dickinson | SK7 | 5.0 |
|  | IFN-γ | FITC | Invitrogen | KSB3 | 5.0 |
|  | TNF-α | PE-Cy | Becton Dickinson | Mab11 | 5.0 |
|  | IL-2 | APC | Invitrogen | MQ1-17H12 | 5.0 |
|  | IL-4 | PE | Invitrogen | 8D4-8 | 5.0 |
|  | IL-17A | Alexa Fluor 647 | Becton Dickinson | N49-653 | 5.0 |
| Monocytes | CD14 | FITC | Becton Dickinson | MφP9 | 5.0 |
|  | IL-10 | PE | Becton Dickinson | JES3-19F1 | 5.0 |
|  | IL-12 | APC | Becton Dickinson | p40/p70 | 5.0 |

**Table S4:** Comparison of individual parameters in groups classified according to PFS (≤6 vs >6 mo) and OS (≤12 vs >12 mo) using Mann-Whitney test.

|  | PFS ≤ 6 mo | PFS > 6 mo |  | OS ≤ 12 mo | OS > 12 mo |  |
| --- | --- | --- | --- | --- | --- | --- |
|  | mean ± SEM (N) | mean ± SEM (N) | *p* value | mean ± SEM (N) | mean ± SEM (N) | *p* value |
| Leu (10^9^/L) | 9.83 ± 0.44 (104) | 9.49 ± 0.57 (98) | 0.22 | 9.73 ± 0.43 (132) | 9.55 ± 0.64 (70) | 0.98 |
| Neu (10^9^/L) | 6.88 ± 0.34 (103) | 6.56 ± 0.41 (98) | 0.29 | 6.81 ± 0.35 (131) | 6.56 ± 0.37 (70) | 1.00 |
| Im gran (10^9^/L) | 0.079 ± 0.010 (103) | 0.070 ± 0.015 (98) | **0.041** | 0.082 ± 0.012 (131) | 0.061 ± 0.011 (70) | 0.22 |
| Ly (10^9^/L) | 1.64 ± 0.08 (103) | 1.7 ± 0.06 (98) | 0.26 | 1.65 ± 0.06 (131) | 1.66 ± 0.07 (70) | 0.49 |
| CD3+ (10^9^/L) | 1.17 ± 0.06 (100) | 1.21 ± 0.05 (96) | 0.31 | 1.18 ± 0.05 (128) | 1.20 ± 0.06 (68) | 0.55 |
| CD3+CD4+ (10^9^/L) | 0.65 ± 0.03 (100) | 0.73 ± 0.04 (96) | 0.12 | 0.68 ± 0.03 (128) | 0.71 ± 0.04 (68) | 0.50 |
| CD3+CD8+ (10^9^/L) | 0.49 ± 0.04 (100) | 0.47 ± 0.03 (96) | 0.64 | 0.49 ± 0.03 (128) | 0.47 ± 0.04 (68) | 0.91 |
| CD3-CD16+CD56+ (10^9^/L) | 0.29 ± 0.02 (100) | 0.30 ± 0.02 (96) | 0.43 | 0.29 ± 0.02 (128) | 0.30 ± 0.02 (68) | 0.68 |
| CD19+ (10^9^/L) | 0.17 ± 0.04 (100) | 0.14 ± 0.01 (96) | 0.47 | 0.16 ± 0.3 (128) | 0.14 ± 0.01 (68) | 0.72 |
| CD3+CD16+CD56+ (10^9^/L) | 0.07 ± 0.01 (100) | 0.05 ± 0.01 (96) | 0.39 | 0.06 ± 0.1 (128) | 0.05 ± 0.01 (68) | 0.64 |
| Mo (10^9^/L) | 0.88 ± 0.04 (106) | 0.77 ± 0.04 (98) | **0.006** | 0.85 ± 0.04 (131) | 0.77 ± 0.05 (70) | **0.042** |
| Eos (10^9^/L) | 0.34 ± 0.10 (103) | 0.44 ± 0.18 (98) | 0.13 | 0.33 ± 0.08 (131) | 0.50 ± 0.25 (70) | 0.14 |
| Bas (10^9^/L) | 0.049 ± 0.003 (103) | 0.570 ± 0.004 (98) | **0.049** | 0.050 ± 0.003 (131) | 0.059 ± 0.005 (70) | 0.06 |
| Treg (10^9^/L) | 0.056 ± 0.003 (88) | 0.072 ± 0.004 (89) | **0.003** | 0.060 ± 0.003 (116) | 0.072 ± 0.005 (61) | **0.033** |
| NLR (-) | 4.95 ± 0.33 (103) | 4.30 ± 0.24 (98) | 0.25 | 4.73 ± 0.26 (131) | 4.44 ± 0.32 (70) | 0.73 |
| C3 (g/L) | 1.44 ± 0.03 (96) | 1.36 ± 0.03 (86) | **0.043** | 1.43 ± 0.02 (120) | 1.37 ± 0.03 (62) | 0.27 |
| C4 (g/L) | 0.32 ± 0.01 (96) | 0.30 ± 0.01 (86) | 0.13 | 0.32 ± 0.01 (120) | 0.29 ± 0.01 (62) | 0.15 |
| CD3+HLA-DR+ (10^9^/L) | 0.21 ± 0.02 (73) | 0.24 ± 0.02 (77) | 0.60 | 0.22 ± 0.01 (87) | 0.23 ± 0.03 (63) | 0.75 |
| CD5+CD19+ (10^9^/L) | 0.066 ± 0.055 (65) | 0.016 ± 0.002 (77) | 0.22 | 0.056 ± 0.045 (80) | 0.017 ± 0.003 (62) | 0.25 |
| CD4+CD45RA (10^9^/L) | 0.22 ± 0.02 (49) | 0.22 ± 0.03 (46) | 0.63 | 0.23 ± 0.02 (51) | 0.21 ± 0.03 (44) | 0.47 |
| IFN gama | 37.25 ± 1.62 (81) | 35.63 ± 1.39 (83) | 0.39 | 36.92 ± 1.38 (107) | 35.52 ± 1.63 (57) | 0.57 |
| TNF alfa | 70.86 ± 1.52 (82) | 72.00 ± 1.40 (83) | 0.57 | 69.98 ± 1.28 (108) | 74.15 ± 1.70 (57) | **0.035** |
| IL-4 | 6.58 ± 0.28 (82) | 6.22 ± 0.25 (82) | 0.29 | 6.31 ± 0.21 (108) | 6.57 ± 0.36 (56) | 0.90 |
| IL-2 | 48.31 ± 1.39 (82) | 49.48 ± 1.41 (81) | 0.62 | 48.78 ± 1.20 (108) | 49.12 ± 1.76 (55) | 0.99 |
| IL-10 | 15.86 ± 0.61 (78) | 16.01 ± 0.62 (80) | 0.87 | 15.78 ± 1.20 (104) | 16.53 ± 0.79 (54) | 0.74 |
| IL-12 | 17.2 ± 0.49 (80) | 16.00 ± 0.64 (78) | 0.14 | 16.42 ± 0.52 (104) | 16.91 ± 0.61 (54) | 0.79 |
| IL-17 | 2.25 ± 0.15 (78) | 2.17 ± 0.12 (81) | 0.85 | 2.23 ± 0.12 (104) | 2.17 ± 0.15 (55) | 0.85 |
| Ery (10^12^/L) | 4.27 ± 0.06 (104) | 4.42 ± 0.05 (98) | 0.07 | 4.32 ± 0.05 (132) | 4.39 ± 0.07 (70) | 0.28 |
| Hb (g/L) | 126.2 ± 1.9 (104) | 132.3 ± 1.7 (98) | **0.009** | 127.6 ± 1.6 (132) | 132.0 ± 2.2 (70) | **0.032** |
| MCV (fl) | 91.6 ± 0.5 (104) | 92.5 ± 0.06 | 0.27 | 91.5 ± 0.4 (132) | 93.1 ± 0.7 (70) | 0.08 |
| MCH (pg) | 29.5 ± 0.2 (104) | 29.9 ± 0.2 (98) | 0.25 | 29.6 ± 0.2 (132) | 30.0 ± 0.3 (70) | 0.22 |
| PLT (10^9^/L) | 323.7 ± 12.5 (104) | 300.0 ± 11.0 (98) | 0.2 | 318.0 ± 10.2 (132) | 301.2 ± 14.4 (70) | 0.24 |
| MPV (fl) | 9.66 ± 0.09 (104) | 9.91 ± 0.04 (98) | **0.036** | 9.68 ± 0.08 (132) | 9.97 ± 0.01 (70) | **0.022** |

**Table S5:** Analysis of PFS and OS using Mantel-Haenszel statistics comparing lower and upper terciles of each category.

|  |  | PFS |  |  |  | OS |  |  |  |
| --- | --- | --- | --- | --- | --- | --- | --- | --- | --- |
|  |  | months | HR | 95% CI | p | months | HR | 95% CI | p |
| CD3+ | lower tercile | 7.0 | 1.66 | 1.06-2.60 | **0.027** | 9.7 | 1.25 | 0.81-1.91 | 0.31 |
|  | upper tercile | 12.0 |  |  |  | 13.2 |  |  |  |
| CD3+CD4+ | lower tercile | 7.9 | 1.48 | 0.95-2.30 | 0.09 | 11.2 | 1.33 | 0.85-2.07 | 0.21 |
| (Th ly) | upper tercile | 11.9 |  |  |  | 13.2 |  |  |  |
| CD3+CD8+ | lower tercile | 8.5 | 1.37 | 0.87-2.16 | 0.17 | 11.2 | 1.22 | 0.79-1.90 | 0.36 |
| (Tc ly) | upper tercile | 12.6 |  |  |  | 13.2 |  |  |  |
| CD3-CD16+CD56+ | lower tercile | 6.8 | 1.12 | 0.72-1.75 | 0.60 | 11.2 | 1.17 | 0.74-1.84 | 0.50 |
| (NK cells) | upper tercile | 8.5 |  |  |  | 13.2 |  |  |  |
| CD19+ | lower tercile | 8.5 | 1.02 | 0.65-1.61 | 0.93 | 8.2 | 1.79 | 1.15-2.79 | **0.010** |
| (B ly) | upper tercile | 9.4 |  |  |  | 17.6 |  |  |  |
| CD3+CD16+CD56+ | lower tercile | 8.7 | 0.83 | 0.53-1.29 | 0.40 | 9.6 | 1.4 | 0.90-2.18 | 0.13 |
| (NKT cells) | upper tercile | 5.4 |  |  |  | 13.3 |  |  |  |
| Treg | lower tercile | **7.9** | **1.97** | **1.22-3.18** | **0.006** | **9.6** | **1.86** | **1.16-2.99** | **0.010** |
|  | upper tercile | **13.8** |  |  |  | **18.3** |  |  |  |
| NLR | lower tercile | 6.1 | 0.76 | 0.49-1.18 | 0.22 | 8.4 | 0.77 | 0.50-1.18 | 0.23 |
|  | upper tercile | 7.9 |  |  |  | 9.6 |  |  |  |
| C3 | lower tercile | **10.6** | **0.55** | **0.35-0.87** | **0.011** | **15.5** | **0.53** | **0.34-0.84** | **0.007** |
|  | upper tercile | **6.1** |  |  |  | **6.6** |  |  |  |
| C4 | lower tercile | 8.2 | 0.95 | 0.60-1.51 | 0.84 | 12.6 | 0.73 | 0.47-1.15 | 0.17 |
|  | upper tercile | 6.6 |  |  |  | 7.5 |  |  |  |
| CD3+HLA-DR+ | lower tercile | 9.1 | 1.14 | 0.70-1.84 | 0.60 | 11.2 | 1.05 | 0.64-1.71 | 0.75 |
|  | upper tercile | 12.6 |  |  |  | 13.6 |  |  |  |
| CD5+CD19+ | lower tercile | 9.1 | 1.16 | 0.72-1.91 | 0.53 | 8.5 | 1.34 | 0.81-2.22 | 0.12 |
|  | upper tercile | 12.6 |  |  |  | 15.5 |  |  |  |
| CD4+CD45RA | lower tercile | 9.1 | 1.23 | 0.69-2.19 | 0.48 | 13.3 | 1.01 | 0.58-1.74 | 0.77 |
|  | upper tercile | 9.4 |  |  |  | 8.0 |  |  |  |
| IFN gama | lower tercile | 9.4 | 0.92 | 0.57-1.49 | 0.73 | 10.9 | 0.87 | 0.54-1.43 | 0.59 |
|  | upper tercile | 7.9 |  |  |  | 13.1 |  |  |  |
| TNF alfa | lower tercile | 6.8 | 1.03 | 0.63-1.69 | 0.90 | 13.2 | 1.03 | 0.62-1.71 | 0.91 |
|  | upper tercile | 10.1 |  |  |  | 14.5 |  |  |  |
| IL-4 | lower tercile | 13.3 | 0.78 | 0.48-1.27 | 0.31 | 20.4 | 0.57 | 0.35-0.93 | **0.024** |
|  | upper tercile | 9.9 |  |  |  | 9.0 |  |  |  |
| IL-2 | lower tercile | 9.2 | 0.89 | 0.55-1.46 | 0.66 | 17.1 | 0.93 | 0.56-1.56 | 0.79 |
|  | upper tercile | 8.5 |  |  |  | 13.1 |  |  |  |
| IL-10 | lower tercile | 10.5 | 1.18 | 0.71-1.93 | 0.53 | 13.6 | 0.94 | 0.57-1.56 | 0.82 |
|  | upper tercile | 9.4 |  |  |  | 12.3 |  |  |  |
| IL-12 | lower tercile | 5.1 | 1.4 | 0.85-2.28 | 0.18 | 8.5 | 1.06 | 0.65-1.73 | 0.81 |
|  | upper tercile | 10.1 |  |  |  | 13.1 |  |  |  |
| IL-17 | lower tercile | 6.6 | 0.98 | 0.61-1.57 | 0.93 | 8.5 | 0.87 | 0.53-1.40 | 0.55 |
|  | upper tercile | 8.8 |  |  |  | 10.9 |  |  |  |
| Leu | lower tercile | 7.9 | 0.82 | 0.54-1.25 | 0.36 | 10.9 | 0.78 | 0.51-1.18 | 0.24 |
|  | upper tercile | 6.1 |  |  |  | 7.3 |  |  |  |
| Neu | lower tercile | 8.2 | 0.7 | 0.46-1.09 | 0.11 | 10.6 | 0.67 | 0.43-1.04 | 0.07 |
|  | upper tercile | 6.5 |  |  |  | 7.3 |  |  |  |
| Ly | lower tercile | 6.0 | 1.42 | 0.92-2.18 | 0.11 | 9.6 | 1.18 | 0.78-1.79 | 0.43 |
|  | upper tercile | 9.2 |  |  |  | 8.5 |  |  |  |
| Mo | lower tercile | **11.9** | **0.61** | **0.40-0.94** | **0.024** | **17.1** | **0.6** | **0.39-0.91** | **0.018** |
|  | upper tercile | **5.1** |  |  |  | **7.3** |  |  |  |
| Eos | lower tercile | **5.4** | **1.98** | **1.27-3.08** | **0.003** | 8.3 | 1.45 | 0.92-2.27 | 0.11 |
|  | upper tercile | **12.8** |  |  |  | 15.2 |  |  |  |
| Bas | lower tercile | 6.8 | 1.18 | 0.77-1.82 | 0.45 | **8.2** | **1.79** | **1.15-2.79** | **0.010** |
|  | upper tercile | 8.7 |  |  |  | **17.6** |  |  |  |
| Im gran | lower tercile | 9.9 | 0.78 | 0.51-1.21 | 0.28 | **13.3** | **0.63** | **0.41-0.96** | **0.033** |
|  | upper tercile | 8.3 |  |  |  | **7.3** |  |  |  |
| Ery | lower tercile | 5.4 | 1.14 | 0.74-1.76 | 0.56 | 8.2 | 1.29 | 0.84-1.98 | 0.24 |
|  | upper tercile | 8.9 |  |  |  | 13.2 |  |  |  |
| Hb | lower tercile | 5.4 | 1.4 | 0.90-2.20 | 0.14 | **6.0** | **1.62** | **1.05-2.51** | **0.029** |
|  | upper tercile | 11.9 |  |  |  | **14.5** |  |  |  |
| MCV | lower tercile | 6.5 | 1.2 | 0.78-1.86 | 0.40 | 8.3 | 1.41 | 0.79-1.88 | 0.37 |
|  | upper tercile | 9.1 |  |  |  | 13.2 |  |  |  |
| MCH | lower tercile | 8.7 | 1.19 | 0.77-1.84 | 0.44 | 8.3 | 0.76 | 0.92-2.19 | 0.11 |
|  | upper tercile | 9.9 |  |  |  | 13.3 |  |  |  |
| PLT | lower tercile | 9.2 | 0.71 | 0.46-1.10 | 0.13 | 13.3 | 0.76 | 0.49-1.17 | 0.21 |
|  | upper tercile | 6.0 |  |  |  | 8.5 |  |  |  |
| MPV | lower tercile | **5.1** | **1.8** | **1.17-2.77** | **0.008** | **7.3** | **2.18** | **1.41-3.35** | **<0.001** |
|  | upper tercile | **10.1** |  |  |  | **15.2** |  |  |  |

**Table S6:** Comparison of individual parameters in groups classified according to the initial response to therapy (CR+PR vs PD, SD vs PD) using Mann-Whitney test.

|  |  | mean ± SEM (N) | *p* value |  |  |  | mean ± SEM (N) | *p* value |
| --- | --- | --- | --- | --- | --- | --- | --- | --- |
| Leu (10^9^/L) | CR+PR | 8.55+0.39 (47) | 0.20 |  | CD3+HLA-DR+ (10^9^/L) | CR+PR | 0.27±0.04 (37) | 0.20 |
|  | SD | 10.14+0.76 (72) | 0.70 |  |  | SD | 0.21±0.03 (52) | 0.92 |
|  | PD | 9.63+0.72 (35) | Ref. |  |  | PD | 0.20±0.02 (26) | Ref. |
| Neu (10^9^/L) | CR+PR | 5.56+0.34 (47) | 0.27 |  | CD5+CD19+ (10^9^/L) | CR+PR | 0.011±0.003 (35) | 0.63 |
|  | SD | 7.06+0.55 (72) | 0.62 |  |  | SD | 0.018±0.003 (49) | 0.29 |
|  | PD | 6.65+0.46 (35) | Ref. |  |  | PD | 0.012±0.003 (24) | Ref. |
| Im gran (10^9^/L) | CR+PR | 0.05 ± 0.01 (47) | **0.008** |  | CD4+CD45RA (10^9^/L) | CR±PR | 0.20±0.03 (17) | 0.89 |
|  | SD | 0.08 ± 0.02 (72) | 0.23 |  |  | SD | 0.23±0.03 (36) | 0.81 |
|  | PD | 0.09 ± 0.02 (35) | Ref. |  |  | PD | 0.22±0.22 (21) | Ref. |
| Ly (10^9^/L) | CR±PR | 1.63±0.09 (47) | 0.96 |  | IFN gama | CR±PR | 37.1±2.2 (40) | 0.61 |
|  | SD | 1.67±0.07 (72) | 0.72 |  |  | SD | 36.5±1.8 (58) | 0.81 |
|  | PD | 1.69±0.13 (35) | Ref. |  |  | PD | 35.4±2.6 (29) | Ref. |
| CD3+ (10^9^/L) | CR±PR | 1.21±0.08 (47) | 0.91 |  | TNF alfa | CR±PR | 71.7±1.7 (40) | 1.00 |
|  | SD | 1.19±0.06 (69) | 0.89 |  |  | SD | 72.2±1.8 (58) | 0.76 |
|  | PD | 1.21±0.11 (34) | Ref. |  |  | PD | 70.7±2.9 (29) | Ref. |
| CD3+CD4+ (10^9^/L) | CR±PR | 0.74±0.08 (47) | 0.88 |  | IL-4 | CR±PR | 6.7±0.4 (40) | 0.88 |
|  | SD | 0.68±0.03 (69) | 0.67 |  |  | SD | 6.0±0.3 (57) | 0.27 |
|  | PD | 0.74±0.06 (34) | Ref. |  |  | PD | 6.6±0.5 (29) | Ref. |
| CD3+CD8+ (10^9^/L) | CR±PR | 0.46±0.04 (47) | 0.89 |  | IL-2 | CR±PR | 51.8±1.8 (39) | 0.48 |
|  | SD | 0.48±0.04 (69) | 0.76 |  |  | SD | 48.1±1.6 (57) | 0.62 |
|  | PD | 0.48±0.06 (34) | Ref. |  |  | PD | 49.1±2.5 (29) | Ref. |
| CD3-CD16+CD56+ (10^9^/L) | CR±PR | 0.29±0.02 (47) | 0.86 |  | IL-10 | CR±PR | 16.6±0.9 (39) | 0.78 |
|  | SD | 0.31±0.02 (69) | 0.49 |  |  | SD | 15.7±0.7 (55) | 0.68 |
|  | PD | 0.29±0.04 (34) | Ref. |  |  | PD | 16.0±1.1 (29) | Ref. |
| CD19+ (10^9^/L) | CR±PR | 0.12±0.01 (47) | 0.37 |  | IL-12 | CR±PR | 17.5±0.7 (39) | 0.52 |
|  | SD | 0.14±0.01 (69) | 0.93 |  |  | SD | 16.7±0.7 (55) | 0.66 |
|  | PD | 0.15±0.02 (34) | Ref. |  |  | PD | 16.2±1.2 (29) | Ref. |
| CD3+CD16+CD56+ (10^9^/L) | CR±PR | 0.043±0.008 (47) | 0.76 |  | IL-17 | CR±PR | 2.3±0.2 (39) | 0.86 |
|  | SD | 0.065±0.013 (69) | 0.71 |  |  | SD | 2.0±0.1 (56) | 0.39 |
|  | PD | 0.048±0.012 (34) | Ref. |  |  | PD | 2.4±0.1 (29) | Ref. |
| Mo (10^9^/L) | CR±PR | 0.68 ± 0.03 (47) | **0.019** |  | Ery (10^12^/L) | CR±PR | 4.40±0.09 (47) | 0.57 |
|  | SD | 0.83 ± 0.06 (72) | 0.42 |  |  | SD | 4.36±0.07 (72) | 0.8 |
|  | PD | 0.89 ± 0.08 (35) | Ref. |  |  | PD | 4.30±0.09 (35) | Ref. |
| Eos (10^9^/L) | CR±PR | 0.23±0.03 (47) | 0.88 |  | Hb (g/L) | CR±PR | 130.9±2.7 (47) | 0.39 |
|  | SD | 0.51±0.25 (72) | 0.34 |  |  | SD | 130.8±2.1 (72) | 0.53 |
|  | PD | 0.34±0.17 (35) | Ref. |  |  | PD | 126.8±2.9 (35) | Ref. |
| Bas (10^9^/L) | CR±PR | 0.046±0.004 (47) | 0.49 |  | MCV (fl) | CR±PR | 92.2±0.8 (47) | 0.93 |
|  | SD | 0.058±0.005 (72) | 0.57 |  |  | SD | 92.5±0.7 (72) | 0.56 |
|  | PD | 0.053±0.005 (35) | Ref. |  |  | PD | 91.6±0.9 (35) | Ref. |
| Treg (10^9^/L) | CR±PR | 0.074±0.006 (44) | 0.77 |  | MCH (pg) | CR±PR | 29.8±0.3 (47) | 0.78 |
|  | SD | 0.064±0.004 (65) | 0.86 |  |  | SD | 30.0±0.3 (72) | 0.6 |
|  | PD | 0.067±0.006 (29) | Ref. |  |  | PD | 29.6±0.4 (35) | Ref. |
| C3 (g/L) | CR±PR | 1.35 ± 0.04 (41) | **0.035** |  | PLT (10^9^/L) | CR±PR | 289.8±15.8 (47) | 0.14 |
|  | SD | 1.38 ± 0.03 (65) | **0.039** |  |  | SD | 311.6±13.5 (72) | 0.35 |
|  | PD | 1.47 ± 0.03 (30) | Ref. |  |  | PD | 346.3±24.8 (35) | Ref. |
| C4 (g/L) | CR±PR | 0.30±0.01 (42) | 0.61 |  | MPV (fl) | CR±PR | 9.8±0.1 (47) | 0.36 |
|  | SD | 0.31±0.01 (64) | 0.91 |  |  | SD | 9.9±0.1 (72) | 0.18 |
|  | PD | 0.31±0.01 (30) | Ref. |  |  | PD | 9.6±0.1 (35) | Ref. |
| NLR | CR±PR | 4.13 ± 0.32 (47) | 0.44 |  |  |  |  |  |
|  | SD | 4.65 ± 0.33 (72) | 0.99 |  |  |  |  |  |
|  | PD | 4.69 ± 0.52 (35) | Ref. |  |  |  |  |  |

| **Table S7:** Comparison of individual parameters patient treated with anti-PD1 only in groups classified according to PFS (≤6 vs >6 mo) using Mann-Whitney test.   \|  \| **anti-PD1 only (N=155)** \| \|  \| **anti-PD1 only in the first line (N=68)** \| \| \| **anti-PD1 only in second and higher line (N=87)** \| \| \| \| --- \| --- \| --- \| --- \| --- \| --- \| --- \| --- \| --- \| --- \| \|  \| PFS ≤ 6 mo \| PFS > 6 mo \|  \| PFS ≤ 6 mo \| PFS > 6 mo \|  \| PFS ≤ 6 mo \| PFS > 6 mo \|  \| \|  \| mean ± SEM (N) \| mean ± SEM (N) \| *p* value \| mean ± SEM (N) \| mean ± SEM (N) \| *p* value \| mean ± SEM (N) \| mean ± SEM (N) \| *p* value \| \| Leu (10^9^/L) \| 9.93 v 0.57 (77) \| 9.62 v 0.70 (78) \| 0.29 \| 11.70 v 0.95 (31) \| 11.54 v 1.40 (37) \| 0.19 \| 8.73 v 0.67 (46) \| 7.89 v 0.26 (41) \| 0.70 \| \| Neu (10^9^/L) \| 6.94 v 0.44 (76) \| 6.58 v 0.49 (78) \| 0.38 \| **8.71 v 0.73 (31)** \| **7.88 v 0.96 (37)** \| **0.046** \| 5.73 v 0.47 (45) \| 5.41 v 0.22 (411 \| 0.59 \| \| Im gran (10^9^/L) \| **0.083 v 0.012 (76)** \| **0.071 v 0.018 (78)** \| **0.020** \| 0.095 v 0.017 (31) \| 0.104 v 0.037 (37) \| 0.08 \| 0.075 v 0.016 (45) \| 0.041 v 0.004 (41) \| 0.10 \| \| Ly (10^9^/L) \| 1.61 v 0.09 (76) \| 1.68 v 0.07 (78) \| 0.22 \| **1.55 v 0.14 (31)** \| **1.82 v 0.10 (37)** \| **0.025** \| 1.65 v 0.11 (45) \| 1.55 v 0.09 (41) \| 0.79 \| \| CD3+ (10^9^/L) \| 1.14 v 0.06 (74) \| 1.22 v 0.06 (76) \| 0.26 \| 1.16 v 0.12 (29) \| 1.33 v 0.09 (36) \| 0.11 \| 1.13 v 0.07 (45) \| 1.12 v 0.08 (40) \| 0.84 \| \| CD3+CD4+ (10^9^/L) \| 0.69 v 0.04 (74) \| 0.72 v 0.04 (76) \| 0.69 \| 0.68 v 0.06 (29) \| 0.78 v 0.06 (36) \| 0.31 \| 0.70 v 0.05 (45) \| 0.66 v 0.05 (40) \| 0.70 \| \| CD3+CD8+ (10^9^/L) \| 0.43 v 0.04 (74) \| 0.49 v 0.03 (76) \| 0.06 \| **0.44 v 0.08 (29)** \| **0.55 v 0.05 (36)** \| **0.015** \| 0.43 v 0.04 (45) \| 0.44 v 0.04 (40) \| 0.88 \| \| CD3-CD16+CD56+ (10^9^/L) \| 0.27 v 0.02 (74) \| 0.30 v 0.02 (76) \| 0.33 \| 0.26 v 0.03 (29) \| 0.33 v 0.04 (36) \| 0.11 \| 0.28 v 0.02 (45) \| 0.27 v 0.02 (40) \| 0.93 \| \| CD19+ (10^9^/L) \| 0.19 v 0.05 (74) \| 0.14 v 0.01 (76) \| 0.87 \| 0.14 v 0.02 (29) \| 0.15 v 0.02 (36 \| 0.32 \| 0.22 v 0.08 (45) \| 0.12 v 0.01 (40) \| 0.33 \| \| CD3+CD16+CD56+ (10^9^/L) \| 0.066 v 0.012 (74) \| 0.051 v 0.008 (76) \| 0.97 \| 0.080 v 0.025 (29) \| 0.056 v 0.015 (36) \| 0.73 \| 0.058 v 0.012 (45) \| 0.047 v 0.007 (40) \| 0.69 \| \| Mo (10^9^/L) \| **0.90 v 0.05 (76)** \| **0.78 v 0.05 (78)** \| **0.011** \| 0.97 v 0.09 (31) \| 0.89 v 0.10 (37) \| 0.14 \| **0.86 v 0.06 (45)** \| **0.68 v 0.04 (41)** \| **0.028** \| \| Eos (10^9^/L) \| **0.38 v 0.14 (76)** \| **0.51 v 0.23 (78)** \| **0.045** \| **0.40 v 0.20 (31)** \| **0.87 v 0.48 (37)** \| **0.050** \| 0.18 v 0.02 (45) \| 0.19 v 0.02 (41) \| 0.36 \| \| Bas (10^9^/L) \| **0.049 v 0.004 (76)** \| **0.059 v 0.004 (78)** \| **0.048** \| 0.054 v 0.006 (31) \| 0.069 v 0.009 (37) \| 0.17 \| 0.046 v 0.005 (45) \| 0.050 v 0.004 (41) \| 0.17 \| \| Treg (10^9^/L) \| **0.060 v 0.003 (64)** \| **0.071 v 0.004 (69)** \| **0.050** \| **0.054 v 0.004 (27)** \| **0.76 v 0.006 (34)** \| **0.013** \| 0.065 v 0.005 (37) \| 0.068 v 0.006 (35) \| 0.72 \| \| NLR (-) \| 5.05 v 0.41 (76) \| 4.34 v 0.28 (78) \| 0.26 \| **6.66 v 0.79 (31)** \| **4.60 v 0.47 (37)** \| **0.007** \| 3.94 v 0.34 (45) \| 4.12 v 0.34 (41) \| 0.52 \| \| C3 (g/L) \| **1.46 v 0.03 (73)** \| **1.34 v 0.03 (68)** \| **0.004** \| 1.45 v 0.05 (29) \| 1.35 v 0.05 (34) \| 0.07 \| **1.47 v 0.04 (44)** \| **1.34 v 0.03 (34)** \| **0.025** \| \| C4 (g/L) \| 0.32 v 0.01 (73) \| 0.29 v 0.01 (67) \| 0.34 \| 0.32 v 0.02 (28) \| 0.30 v 0.01 (33) \| 0.96 \| 0.32 v 0.01 (45) \| 0.29 v 0.01 (34) \| 0.20 \| \| CD3+HLA-DR+ (10^9^/L) \| 0.18 v 0.02 (55) \| 0.25 v 0.03 (63) \| 0.20 \| 0.21 v 0.04 (18) \| 0.27 v 0.04 (29) \| 0.42 \| 0.17 v 0.02 (37) \| 0.23 v 0.03 (34) \| 0.42 \| \| CD5+CD19+ (10^9^/L) \| 0.085 v 0.073 (49) \| 0.014 v 0.003 (63) \| 0.85 \| 0.016 v 0.006 (14) \| 0.018 v 0.005 (29) \| 0.91 \| 0.113 v 0.102 (35) \| 0.010 v 0.002 (34) \| 1.00 \| \| CD4+CD45RA (10^9^/L) \| 0.24 v 0.03 (42) \| 0.22 v 0.03 (45) \| 0.51 \| 0.24 v 0.04 (10) \| 0.27 v 0.04 (18) \| 0.79 \| 0.24 v 0.03 (32) \| 0.19 v 0.04 (27) \| 0.18 \| \| IFN gama \| 34.18 v 1.86 (58) \| 36.20 v 1.74 (62) \| 0.38 \| **31.49 v 3.01 (22)** \| **40.42 v 2.81 (29)** \| **0.023** \| 35.82 v 2.35 (36) \| 32.49 v 1.97 (33) \| 0.38 \| \| TNF alfa \| 70.03 v 1.93 (59) \| 73.85 v 1.58 (62) \| 0.14 \| **63.40 v 3.28 (23)** \| **73.35 v 2.07 (29)** \| **0.020** \| 74.26 v 2.10 (36) \| 74.29 v 2.37 (33) \| 0.81 \| \| IL-4 \| 6.62 v 0.36 (59) \| 6.20 v 0.30 (61) \| 0.32 \| 6.42 v 0.52 (23) \| 5.86 v 0.45 (29) \| 0.16 \| 6.74 v 0.48 (36) \| 6.51 v 0.39 (32) \| 0.89 \| \| IL-2 \| 49.28 v 1.57 (59) \| 48.18 v 1.70 (61) \| 0.44 \| 47.46 v 3.17 (23) \| 49.25 v 2.37 (29) \| 0.85 \| 50.45 v 1.61 (36) \| 47.2 v 2.45 (32) \| 0.15 \| \| IL-10 \| 15.82 v 0.71 (56) \| 16.19 v 0.71 (59) \| 0.96 \| 15.61 v 1.40 (22) \| 15.39 v 0.95 (27) \| 0.77 \| 15.95 v 0.76 (34) \| 16.86 v 1.04 (32) \| 0.87 \| \| IL-12 \| 15.78 v 0.78 (56) \| 17.20 v 0.56 (59) \| 0.13 \| 14.22 v 1.09 (22) \| 16.06 v 0.84 (27) \| 0.28 \| 16.79 v 1.05 (34) \| 18.17 v 0.71 (32) \| 0.25 \| \| IL-17 \| 2.37 v 0.18 (56) \| 2.21 v 0.14 (60) \| 0.79 \| 2.04 v 0.20 (22) \| 2.13 v 0.24 (27) \| 0.80 \| 2.59 v 0.27 (34) \| 2.26 v 0.16 (33) \| 0.60 \| \| Ery (10^12^/L) \| 4.22 v 0.07 (77) \| 4.35 v 0.06 (78) \| 0.13 \| **4.18 v 0.13 (31)** \| **4.48 v 0.09 (37)** \| **0.030** \| 4.24 v 0.09 (46) \| 4.24 v 0.08 (41) \| 0.97 \| \| Hb (g/L) \| **123.9 v 2.3 (77)** \| **131.2 v 1.9 (78)** \| **0.010** \| **123.4 v 3.9 (31)** \| **131.7 v 2.7 (37)** \| **0.037** \| 124.3 v 2.75 (46) \| 130.8 v 2.63 (41) \| 0.09 \| \| MCV (fl) \| 91.4 v 0.6 (77) \| 93.0 v 0.6 (78) \| 0.08 \| 91.1 v 0.8 (31) \| 90.9 v 0.8 (37) \| 0.98 \| **91.6 v 0.8 (46)** \| **94.9 v 0.9 (41)** \| **0.013** \| \| MCH (pg) \| 9.44 v 0.23 (77) \| 30.08 v 0.26 (78) \| 0.07 \| 29.6 v 0.3 (31) \| 29.5 v 0.3 (37) \| 0.96 \| **29.4 v 0.3 (46)** \| **30.6 v 0.4 (41)** \| **0.014** \| \| PLT (10^9^/L) \| 329.4 v 14.3 (77) \| 296.8 v 12.2 (78) \| 0.10 \| 342.0 v 21.8 (31) \| 319.8 v 19.1 (37) \| 0.44 \| 320.8 v 19.0 (46) \| 276.1 v 15.1 (41) \| 0.11 \| \| MPV (fl) \| **9.64 v 0.10 (77)** \| **9.94 v 0.10 (78)** \| **0.028** \| 9.64 v 0.18 (31) \| 9.91 v 0.15 (37) \| 0.20 \| 9.64 v 0.12 (46) \| 9.95 v 0.14 (41) \| 0.07 \|   **Table S8:** Comparison of individual parameters patient treated with anti-PD1 only in groups classified according to PFS (≤6 vs >6 mo) using Mann-Whitney test.   \|  \| **anti-PD1 only (N=155)** \| \|  \| **anti-PD1 only in the first line (N=68)** \| \| \| **anti-PD1 only in second and higher line (N=87)** \| \| \| \| --- \| --- \| --- \| --- \| --- \| --- \| --- \| --- \| --- \| --- \| \|  \| OS ≤ 12 mo \| OS > 12 mo \|  \| OS ≤ 12 mo \| OS > 12 mo \|  \| OS ≤ 12 mo \| OS > 12 mo \|  \| \|  \| mean ± SEM (N) \| mean ± SEM (N) \| *p* value \| mean ± SEM (N) \| mean ± SEM (N) \| *p* value \| mean ± SEM (N) \| mean ± SEM (N) \| *p* value \| \| Leu (10^9^/L) \| 10.03 v 0.58 (94) \| 9.37 v 0.72 (61) \| 0.56 \| 11.99 v 1.00 (42) \| 11.01 v 1.6 (26) \| 0.17 \| 8.45 v 0.60 (52) \| 8.16 v 0.28 (35) \| 0.39 \| \| Neu (10^9^/L) \| 7.03 v 0.47 (93) \| 6.36 v 0.40 (61) \| 0.58 \| 8.81 v 0.85 (42) \| 7.37 v 0.85 (26) \| 0.07 \| 5.56 v 0.41 (51) \| 5.61 v 0.25 (35) \| 0.15 \| \| Im gran (10^9^/L) \| 0.086 v 0.016 (93) \| 0.063 v 0.013 (61) \| 0.16 \| 0.106 v 0.030 (42) \| 0.091 v 0.030 (26) \| 0.43 \| 0.070 v 0.014 (51) \| 0.043 v 0.004 (35) \| 0.25 \| \| Ly (10^9^/L) \| 1.65 v 0.08 (93) \| 1.64 v 0.07 (61) \| 0.42 \| 1.69 v 0.12 (42) \| 1.71 v 0.11 (26) \| 0.44 \| 1.61 v 0.11 (51) \| 1.60 v 0.09 (35) \| 0.65 \| \| CD3+ (10^9^/L) \| 1.17 v 0.06 (91) \| 1.20 v 0.06 (59) \| 0.38 \| 1.25 v 0.10 (40) \| 1.25 v 0.10 (25) \| 0.66 \| 1.10 v 0.07 (51) \| 1.16 v 0.08 (34) \| 0.48 \| \| CD3+CD4+ (10^9^/L) \| 0.70 v 0.04 (91) \| 0.71 v 0.04 (59) \| 0.62 \| 0.74 v 0.05 (40) \| 0.72 v 0.06 (25) \| 0.83 \| 0.66 v 0.05 (51) \| 0.71 v 0.06 (34) \| 0.45 \| \| CD3+CD8+ (10^9^/L) \| 0.45 v 0.03 (91) \| 0.48 v 0.04 (59) \| 0.42 \| 0.48 v 0.06 (40) \| 0.54 v 0.07 (25) \| 0.36 \| 0.43 v 0.04 (51) \| 0.45 v 0.05 (34) \| 0.79 \| \| CD3-CD16+CD56+ (10^9^/L) \| 0.28 v 0.02 (91) \| 0.28 v 0.02 (59) \| 0.67 \| 0.30 v 0.03 (40) \| 0.30 v 0.04 (25) \| 0.59 \| 0.28 v 0.02 (51) \| 0.28 v 0.03 (34) \| 0.86 \| \| CD19+ (10^9^/L) \| 0.18 v 0.04 (91) \| 0.13 v 0.01 (59) \| 0.80 \| 0.15 v 0.02 (40) \| 0.13 v 0.02 (25) \| 0.80 \| 0.20 v 0.07 (51) \| 0.14 v 0.02 (34) \| 0.94 \| \| CD3+CD16+CD56+ (10^9^/L) \| 0.06 v 0.04 (91) \| 0.05 v 0.01 (59) \| 0.94 \| 0.077 v 0.020 (40) \| 0.051 v 0.015 (25) \| 0.42 \| 0.054 v 0.011 (51) \| 0.051 v 0.008 (34) \| 0.45 \| \| Mo (10^9^/L) \| **0.89 v 0.04 (93)** \| **0.78 v 0.06 (61)** \| **0.022** \| **0.97 v 0.08 (42)** \| **0.87 v 0.13 (26)** \| **0.041** \| 0.82 v 0.05 (51) \| 0.071 v 0.04 (35) \| 0.26 \| \| Eos (10^9^/L) \| 0.30 v 0.07 (93) \| 0.54 v 0.29 (61) \| 0.22 \| 0.45 v 0.15 (42) \| 0.99 v 0.67 (26) \| 0.50 \| 0.18 v 0.02 (51) \| 0.20 v 0.03 (35) \| 0.23 \| \| Bas (10^9^/L) \| 0.051 v 0.003 (93) \| 0.059 v 0.005 (61) \| 0.17 \| 0.058 v 0.005 (42) \| 0.069 v 0.012 (26) \| 0.43 \| 0.046 v 0.004 (51) \| 0.051 v 0.004 (35) \| 0.19 \| \| Treg (10^9^/L) \| 0.062 v 0.003 (81) \| 0.071 v 0.004 (52) \| 0.11 \| 0.062 v 0.005 (37) \| 0.071 v 0.006 (24) \| 0.17 \| 0.063 v 0.005 (43) \| 0.072 v 0.007 (28) \| 0.36 \| \| NLR (-) \| 4.92 v 0.34 (93) \| 4.35 v 0.35 (61) \| 0.32 \| 5.98 v 0.59 (42) \| 4.82 v 0.69 (26) \| 0.07 \| 4.05 v 0.33 (51) \| 4.00 v 0.33 (35) \| 0.68 \| \| C3 (g/L) \| 1.43 v 0.03 (87) \| 1.36 v 0.03 (54) \| 0.16 \| 1.41 v 0.05 (38) \| 1.37 v 0.05 (25) \| 0.84 \| 1.45 v 0.04 (49) \| 1.35 v 0.03 (29) \| 0.08 \| \| C4 (g/L) \| 0.32 v 0.01 (87) \| 0.37 v 0.08 (54) \| 0.29 \| 0.31 v 0.02 (37) \| 0.31 v 0.01 (24) \| 0.72 \| **0.32 v 0.01 (50)** \| **0.28 v 0.01 (29)** \| **0.047** \| \| CD3+HLA-DR+ (10^9^/L) \| 0.20 v 0.02 (64) \| 0.24 v 0.03 (54) \| 0.57 \| 0.24 v 0.04 (23) \| 0.25 v 0.05 (24) \| 0.68 \| 0.17 v 0.01 (41) \| 0.23 v 0.04 (30) \| 0.33 \| \| CD5+CD19+ (10^9^/L) \| 0.072 v 0.006 (59) \| 0.015 v 0.003 (53) \| 0.84 \| 0.014 v 0.004 (19) \| 0.020 v 0.006 (24) \| 0.80 \| 0.100 v 0.089 (40) \| 0.011 v 0.003 (29) \| 0.98 \| \| CD4+CD45RA (10^9^/L) \| 0.24 v 0.03 (46) \| 0.22 v 0.03 (41) \| 0.63 \| 0.27 v 0.03 (12) \| 0.24 v 0.04 (16) \| 0.31 \| 0.22 v 0.03 (34) \| 0.21 v 0.04 (25) \| 0.84 \| \| IFN gama \| 39.61 v 1.74 (72) \| 36.14 v 1.81 (48) \| 0.47 \| 35.51 v 2.69 (31) \| 38.22 v 3.55 (20) \| 0.60 \| 33.93 v 2.31 (41) \| 34.66 v 1.80 (28) \| 0.60 \| \| TNF alfa \| 70.25 v 1.64 (73) \| 74.62 v 1.86 (48) \| 0.07 \| 65.74 v 0.42 (32) \| 74.08 v 2.44 (20) \| 0.06 \| 73.78 v 1.90 (41) \| 75.01 v 2.71 (28) \| 0.43 \| \| IL-4 \| 6.24 v 0.28 (73) \| 6.66 v 0.40 (47) \| 0.61 \| 5.91 v 0.42 (32) \| 6.44 v 0.59 (20) \| 0.81 \| 6.50 v 0.37 (41) \| 6.83 v 0.55 (27) \| 0.76 \| \| IL-2 \| 48.87 v 1.43 (73) \| 48.49 v 1.97 (47) \| 0.69 \| 48.07 v 2.57 (32) \| 49.08 v 2.87 (20) \| 0.94 \| 49.49 v 1.58 (41) \| 48.05 v 2.73 (27) \| 0.65 \| \| IL-10 \| 15.75 v 0.62 (70) \| 16.41 v 0.85 (45) \| 0.96 \| 15.84 v 1.09 (31) \| 14.88 v 1.17 (18) \| 0.38 \| 15.67 v 0.71 (39) \| 17.43 v 1.16 (27) \| 0.47 \| \| IL-12 \| 16.31 v 0.66 (70) \| 16.83 v 0.67 (45) \| 0.91 \| 15.21 v 0.97 (31) \| 15.27 v 0.83 (18) \| 0.51 \| 17.18 v 0.88 (39) \| 17.87 v 0.94 (27) \| 0.57 \| \| IL-17 \| 2.37 v 0.15 (70) \| 2.15 v 0.16 (46) \| 0.33 \| 2.16 v 0.18 (31) \| 1.97 v 0.30 (18) \| 0.36 \| 2.54 v 0.24 (39) \| 2.27 v 0.09 (28) \| 0.55 \| \| Ery (10^12^/L) \| 4.25 v 0.06 (94) \| 4.34 v 0.07 (61) \| 0.24 \| 4.27 v 0.10 (42) \| 4.46 v 0.13 (26) \| 0.14 \| 4.23 v 0.08 (52) \| 4.26 v 0.09 (35) \| 0.71 \| \| Hb (g/L) \| **125.3 v 1.9 (94)** \| **131.1 v 2.3 (61)** \| **0.025** \| 126.3 v 2.93 (42) \| 130.5 v 3.91 (26) \| 0.18 \| 124.5 v 2.52 (52) \| 131.7 v 2.9 (35) \| 0.07 \| \| MCV (fl) \| 91.5 v 0.5 (94) \| 93.3 v 0.8 (61) \| 0.08 \| 91.2 v 0.7 (42) \| 90.7 v 1.1 (26) \| 0.75 \| **91.8 v 0.7 (52)** \| **95.3 v 0.9 (35)** \| **0.014** \| \| MCH (pg) \| 29.5 v 0.2 (94) \| 30.1 v 0.3 (61 \| 0.17 \| 29.6 v 0.3 (42) \| 29.3 v 0.4 (26) \| 0.69 \| **29.5 v 0.31 (52)** \| **30.7 v 0.4 (35)** \| **0.041** \| \| PLT (10^9^/L) \| 323.4 v 12.1 (94) \| 296.9 v 15.1 (61) \| 0.13 \| 335.1 v 17.1 (42) \| 321.6 v 25.7 (26) \| 0.50 \| 314.0 v 16.9 (52) \| 278.6 v 17.9 (35) \| 0.20 \| \| MPV (fl) \| **9.68 v 0.09 (94)** \| **9.96 v 0.12 (61)** \| **0.043** \| 9.66 v 0.14 (42) \| 9.99 v 0.20 (26) \| 0.19 \| 9.69 v 0.12 (52) \| 9.94 v 0.15 (35) \| 0.11 \|   **Table S9:** Comparison of the individual parameters before and after therapy according to PFS (≤6 vs >6 mo) and OS (≤12 vs >12 mo) using Mann-Whitney test. | | | | | | |
| --- | --- | --- | --- | --- | --- | --- | --- | --- | --- | --- | --- | --- | --- | --- | --- | --- | --- | --- | --- | --- | --- | --- | --- | --- | --- | --- | --- | --- | --- | --- | --- | --- | --- | --- | --- | --- | --- | --- | --- | --- | --- | --- | --- | --- | --- | --- | --- | --- | --- | --- | --- | --- | --- | --- | --- | --- | --- | --- | --- | --- | --- | --- | --- | --- | --- | --- | --- | --- | --- | --- | --- | --- | --- | --- | --- | --- | --- | --- | --- | --- | --- | --- | --- | --- | --- | --- | --- | --- | --- | --- | --- | --- | --- | --- | --- | --- | --- | --- | --- | --- | --- | --- | --- | --- | --- | --- | --- | --- | --- | --- | --- | --- | --- | --- | --- | --- | --- | --- | --- | --- | --- | --- | --- | --- | --- | --- | --- | --- | --- | --- | --- | --- | --- | --- | --- | --- | --- | --- | --- | --- | --- | --- | --- | --- | --- | --- | --- | --- | --- | --- | --- | --- | --- | --- | --- | --- | --- | --- | --- | --- | --- | --- | --- | --- | --- | --- | --- | --- | --- | --- | --- | --- | --- | --- | --- | --- | --- | --- | --- | --- | --- | --- | --- | --- | --- | --- | --- | --- | --- | --- | --- | --- | --- | --- | --- | --- | --- | --- | --- | --- | --- | --- | --- | --- | --- | --- | --- | --- | --- | --- | --- | --- | --- | --- | --- | --- | --- | --- | --- | --- | --- | --- | --- | --- | --- | --- | --- | --- | --- | --- | --- | --- | --- | --- | --- | --- | --- | --- | --- | --- | --- | --- | --- | --- | --- | --- | --- | --- | --- | --- | --- | --- | --- | --- | --- | --- | --- | --- | --- | --- | --- | --- | --- | --- | --- | --- | --- | --- | --- | --- | --- | --- | --- | --- | --- | --- | --- | --- | --- | --- | --- | --- | --- | --- | --- | --- | --- | --- | --- | --- | --- | --- | --- | --- | --- | --- | --- | --- | --- | --- | --- | --- | --- | --- | --- | --- | --- | --- | --- | --- | --- | --- | --- | --- | --- | --- | --- | --- | --- | --- | --- | --- | --- | --- | --- | --- | --- | --- | --- | --- | --- | --- | --- | --- | --- | --- | --- | --- | --- | --- | --- | --- | --- | --- | --- | --- | --- | --- | --- | --- | --- | --- | --- | --- | --- | --- | --- | --- | --- | --- | --- | --- | --- | --- | --- | --- | --- | --- | --- | --- | --- | --- | --- | --- | --- | --- | --- | --- | --- | --- | --- | --- | --- | --- | --- | --- | --- | --- | --- | --- | --- | --- | --- | --- | --- | --- | --- | --- | --- | --- | --- | --- | --- | --- | --- | --- | --- | --- | --- | --- | --- | --- | --- | --- | --- | --- | --- | --- | --- | --- | --- | --- | --- | --- | --- | --- | --- | --- | --- | --- | --- | --- | --- | --- | --- | --- | --- | --- | --- | --- | --- | --- | --- | --- | --- | --- | --- | --- | --- | --- | --- | --- | --- | --- | --- | --- | --- | --- | --- | --- | --- | --- | --- | --- | --- | --- | --- | --- | --- | --- | --- | --- | --- | --- | --- | --- | --- | --- | --- | --- | --- | --- | --- | --- | --- | --- | --- | --- | --- | --- | --- | --- | --- | --- | --- | --- | --- | --- | --- | --- | --- | --- | --- | --- | --- | --- | --- | --- | --- | --- | --- | --- | --- | --- | --- | --- | --- | --- | --- | --- | --- | --- | --- | --- | --- | --- | --- | --- | --- | --- | --- | --- | --- | --- | --- | --- | --- | --- | --- | --- | --- | --- | --- | --- | --- | --- | --- | --- | --- | --- | --- | --- | --- | --- | --- | --- | --- | --- | --- | --- | --- | --- | --- | --- | --- | --- | --- | --- | --- | --- | --- | --- | --- | --- | --- | --- | --- | --- | --- | --- | --- | --- | --- | --- | --- | --- | --- | --- | --- | --- | --- | --- | --- | --- | --- | --- | --- | --- | --- | --- | --- | --- | --- | --- | --- | --- | --- | --- | --- | --- | --- | --- | --- | --- | --- | --- | --- | --- | --- | --- | --- | --- | --- | --- | --- | --- | --- | --- | --- | --- | --- | --- | --- | --- | --- | --- | --- | --- | --- | --- | --- | --- | --- | --- | --- | --- | --- | --- | --- | --- | --- | --- | --- | --- | --- | --- | --- | --- | --- | --- | --- | --- | --- | --- | --- | --- | --- | --- | --- | --- | --- | --- | --- | --- | --- | --- | --- | --- | --- | --- | --- | --- | --- | --- | --- | --- | --- | --- | --- | --- | --- | --- | --- | --- | --- | --- | --- | --- | --- | --- | --- | --- | --- | --- | --- | --- | --- | --- | --- | --- | --- | --- | --- | --- | --- | --- | --- | --- | --- | --- | --- | --- | --- | --- | --- | --- |
|  | **PFS ≤ 6 mo before** | **PFS ≤ 6 mo after** |  | **OS ≤ 12 mo before** | **OS ≤ 12 mo after** |  |
|  | **mean ± SEM (N)** | **mean ± SEM (N)** | ***p* value** | **mean ± SEM (N)** | **mean ± SEM (N)** | ***p* value** |
| Leu (10^9^/L) | 9.83 ± 0.44 (104) | 10.06 ± 0.57 (79) | 0.80 | 9.73 ± 0.43 (132) | 9.51 ± 0.46 (105) | 0.42 |
| Neu (10^9^/L) | 6.88 ± 0.34 (103) | 7.38 ± 0.50 (79) | 1.00 | 6.81 ± 0.35 (131) | 6.78 ± 0.40 (105) | 0.37 |
| Im gran (10^9^/L) | 0.079 ± 0.010 (103) | 0.108 ± 0.019 (79) | 0.42 | 0.082 ± 0.012 (131) | 0.097 ± 0.015 (105) | 0.15 |
| Ly (10^9^/L) | 1.64 ± 0.08 (103) | 1.53 ± 0.12 (79) | **0.027** | 1.65 ± 0.06 (131) | 1.61 ± 0.10 (105) | 0.58 |
| Mo (10^9^/L) | 0.88 ± 0.04 (106) | 0.87 ± 0.05 (79) | 0.53 | 0.85 ± 0.04 (131) | 0.84 ± 0.04 (105) | 0.24 |
| Eos (10^9^/L) | 0.34 ± 0.10 (103) | 0.21 ± 0.05 (79) | 0.29 | 0.33 ± 0.08 (131) | 0.22 ± 0.04 (105) | 0.31 |
| Bas (10^9^/L) | 0.049 ± 0.003 (103) | 0.050 ± 0.006 (79) | 0.17 | 0.050 ± 0.003 (131) | 0.050 ± 0.005 (105) | 0.81 |
| NLR (no) | 4.95 ± 0.33 (103) | 6.37 ± 0.64 (79) | 0.25 | 4.73 ± 0.26 (131) | 5.65 ± 0.52 (105) | 0.96 |
| Ery (10^12^/L) | 4.27 ± 0.06 (104) | 4.06 ± 0.08 (79) | 0.05 | 4.32 ± 0.05 (132) | 4.13 ± 0.06 (105) | 0.05 |
| Hb (g/L) | 126.2 ± 1.9 (104) | 117.5 ± 2.3 (79) | **0.005** | 127.6 ± 1.6 (132) | 120.6 ± 1.9 (105) | **0.015** |
| MCV (fl) | 91.6 ± 0.5 (104) | 90.4 ± 0.7 (79) | 0.18 | 91.5 ± 0.4 (132) | 90.6 ± 0.6 (105) | 0.23 |
| MCH (pg) | 29.5 ± 0.2 (104) | 29.1 ± 0.3 (79) | 0.13 | 29.6 ± 0.2 (132) | 29.4 ± 0.2 (105) | 0.42 |
| PLT (10^9^/L) | 323.7 ± 12.5 (104) | 311.3 ± 15.1 (79) | 0.45 | 318.0 ± 10.2 (132) | 301.8 ± 12.0 (105) | 0.22 |
| MPV (fl) | 9.66 ± 0.09 (104) | 9.67 ± 0.12 (79) | 0.94 | 9.68 ± 0.08 (132) | 9.62 ± 0.10 (105) | 0.59 |
|  | **PFS > 6 mo before** | **PFS > 6 mo after** |  | **OS > 12 mo before** | **OS > 12 mo after** |  |
|  | **mean ± SEM (N)** | **mean ± SEM (N)** | ***p* value** | **mean ± SEM (N)** | **mean ± SEM (N)** | ***p* value** |
| Leu (10^9^/L) | 9.49 ± 0.57 (98) | 8.24 ± 0.31 (93) | 0.06 | 9.55 ± 0.64 (70) | 8.39 ± 0.39 (67) | 0.08 |
| Neu (10^9^/L) | 6.56 ± 0.41 (98) | 5.45 ± 0.28 (93) | **0.003** | 6.56 ± 0.37 (70) | 5.64 ± 0.35 (67) | **0.010** |
| Im gran (10^9^/L) | 0.070 ± 0.015 (98) | 0.059 ± 0.008 (93) | 0.67 | 0.061 ± 0.011 (70) | 0.059 ± 0.009 (67) | 0.86 |
| Ly (10^9^/L) | 1.7 ± 0.06 (98) | 1.74 ± 0.07 (93) | 0.80 | 1.66 ± 0.07 (70) | 1.70 ± 0.08 (67) | 0.99 |
| Mo (10^9^/L) | 0.77 ± 0.04 (98) | 0.73 ± 0.03 (93) | 0.51 | 0.77 ± 0.05 (70) | 0.73 ± 0.03 (67) | 0.71 |
| Eos (10^9^/L) | 0.44 ± 0.18 (98) | 0.25 ± 0.03 (93) | 0.52 | 0.50 ± 0.25 (70) | 0.26 ± 0.04 (67) | 0.35 |
| Bas (10^9^/L) | 0.570 ± 0.004 (98) | 0.051 ± 0.003 (93) | 0.59 | 0.059 ± 0.005 (70) | 0.051 ± 0.003 (67) | 0.88 |
| NLR (no) | 4.30 ± 0.24 (98) | 3.65 ± 0.27 (93) | **0.001** | 4.44 ± 0.32 (70) | 3.72 ± 0.27 (67) | **0.014** |
| Ery (10^12^/L) | 4.42 ± 0.05 (98) | 4.37 ± 0.06 (93) | 0.70 | 4.39 ± 0.07 (70) | 4.40 ± 0.08 (67) | 0.92 |
| Hb (g/L) | 132.3 ± 1.7 (98) | 131.3 ± 1.8 (93) | 0.74 | 132.0 ± 2.2 (70) | 131.8 ± 2.3 (67) | 0.90 |
| MCV (fl) | 92.5 ± 0.06 | 92.6 ± 0.6 (93) | 0.95 | 93.1 ± 0.7 (70) | 93.1 ± 0.8 (67) | 1.00 |
| MCH (pg) | 29.9 ± 0.2 (98) | 30.2 ± 0.3 (93) | 0.58 | 30.0 ± 0.3 (70) | 30.1 ± 0.3 (67) | 0.96 |
| PLT (10^9^/L) | 300.0 ± 11.0 (98) | 274.4 ± 11.2 (93) | 0.05 | 301.2 ± 14.4 (70) | 273.9 ± 14.6 (67) | 0.11 |
| MPV (fl) | 9.91 ± 0.04 (98) | 9.87 ± 0.11 (93) | 0.77 | 9.97 ± 0.01 (70) | 10.03 ± 0.13 (67) | 0.83 |
